# Advancing the modernization of traditional Chinese medicine through artificial intelligence and multimodal data integration

**DOI:** 10.1186/s13020-025-01194-y

**Published:** 2026-01-26

**Authors:** Pengfei Guo, Mengmeng Jiang, Shengquan Hu, Qianqian Jiang, Limin Li, Junhong Wu, Yucui Ma, Zhengzhi Wu

**Affiliations:** 1 The First Affiliated Hospital of Shenzhen University, Shenzhen, 518035 China; 2 Wu Zhengzhi Academician Workstation, NingBo College of Health Sciences, Ningbo, 315800 China; 3 Hunan University of Traditional Chinese Medicine, Changsha, 410073 China

**Keywords:** Traditional Chinese Medicine, Artificial intelligence (AI), Knowledge graph, Natural language processing, Large language models

## Abstract

Traditional Chinese Medicine (TCM) is a valuable medical treasure trove that not only demonstrated unique advantages in treating complex and refractory diseases but also left behind a rich legacy of ancient texts and valuable evidence-based medical data based on its human experience for future generations. Nevertheless, the extensive data within TCM has been plagued by challenges, including inadequate data standardization, inconsistent data quality, limited data structuring, and obstacles in interdisciplinary integration. Recent advancements in artificial intelligence (AI) techniques have markedly improved the efficiency and effectiveness with which multimodal data in TCM, including machine learning (ML), deep learning (DL), knowledge graphs (KG), and natural language processing (NLP), particularly large language models (LLMs). These advancements have facilitated more precise data analysis, enhanced clinical decision-making, and improved research outcomes in TCM, such as target discovery, virtual screening of natural products (NPs), symptom differentiation and auxiliary prescription. This article presents a comprehensive review of the progress in applying AI across four dimensions: multiscale data in TCM, TCM research and development, TCM diagnosis and treatment, and LLMs. In summary, the application of AI technology in the modernization of TCM is expected to motivate researchers to achieve a deeper understanding of state-of-the-art applications in data-driven TCM complex systems, fundamental scientific research, and precision medicine, thereby bringing more opportunities and innovations for the modernization of TCM.

**Supplementary Information:**

The online version contains supplementary material available at 10.1186/s13020-025-01194-y.

## Introduction

For centuries, TCM has been a traditional system of ethnopharmacology, rich in effective natural resources, and encompasses a wealth of valuable human usage experience. This includes specialized ancient TCM literature, case studies by renowned elderly TCM practitioners, medical record materials, journal papers, summaries of clinical experience, and traditional processing techniques of Chinese herbal medicines [1, 2]. The complex data types in TCM, with their multi-dimensional perspectives, extensive sample sizes, and holistic systemic nature, are progressively becoming a key avenue for computational cognition of the TCM domain. These characteristics facilitated the digital and quantitative analysis of TCM, enabling deeper insights and more precise understanding.

In recent years, the rapid advancement of big biomedical data technologies has provided a solid foundation for the standardization of complex data in TCM, such as high-throughput methods, multi-omics approaches, and network pharmacology (NPM). For example, Fan et al. comprehensively reviewed and summarized the 60-year history of multi-level databases in TCM systems pharmacology, encompassing formula databases, herb databases, component databases, target and target-related biofunction databases, and phenotype databases and *ZHENG* databases, underscore the significant and rapid progress in the development of TCM data [3]. The integration of intelligent technologies with TCM big data revealed vast prospects and potential. For instance, in formula recommendation, ETCM could integrate patient constitution and symptoms to intelligently recommend personalized TCM prescriptions, thereby enhancing the precision and effectiveness of TCM treatments [4]. In terms of herbal quality control, the simplification functionality in TCMSID, designed to identify key ingredients in each herb or formula, might restrict comprehensive investigation into the detailed mechanisms of action of these components within TCM databases [5].

Simultaneously, with the continuous emergence and rapid development of new technologies such as ChatGPT, DeepSeek and AlphaFold3, AI-empowered modernization of TCM is emerging as the central intellectual productivity driving the development and advancement of TCM. It paved the way for substantial progress in AI technologies, enabling more sophisticated data mining techniques, enhanced construction of KG, advanced NLP, and improved reasoning capabilities through ML and DL algorithms. This integration not only enriched the AI-driven analysis of TCM data but also promoted cross-disciplinary research and innovation in healthcare, such as accelerated NPs discovery [6], target validation [7], enhanced precision medicine [8], support for clinical decision-making [9], etc. For example, utilizing an artificial neural network (ANN)-based DL approach, Wang et al. developed an ontology-based side-effect prediction framework (OSPF) to offer well-grounded recommendations for selecting appropriate TCM prescriptions as a complementary treatment for COVID-19 [10]. Cheng et al. proposed an S-TextBLCNN model for the efficacy classification of TCM prescriptions by leveraging NLP to learn and implement the quantitative expression of different TCM concepts [11]. In addition, leveraging extensive TCM datasets and advanced algorithmic models, LLMs were capable of extracting and generating TCM knowledge, facilitating the organic integration of fundamental core data in herbal medicine research with the development and clinical application of TCM, such as TCMChat [12], Lindan [13] and MedChatZH [14]. For instance, Dai et al. constructed a generative LLM TCMChat by compiling a customized collection of six scenarios of Chinese medicine, including TCM knowledgebase, choice question, reading comprehension, entity extraction, medical case diagnosis, and herb or formula recommendation. After pre-training (PT) and supervised fine-tuning (SFT), their research also found that the LLM model exhibited excellent performance based on Baichuan2-7B-Chat foundation model [12]. Therefore, the availability of multimodal data for TCM also encourages interdisciplinary collaboration between TCM and AI technologies [15, 16].

**Fig. 1:**
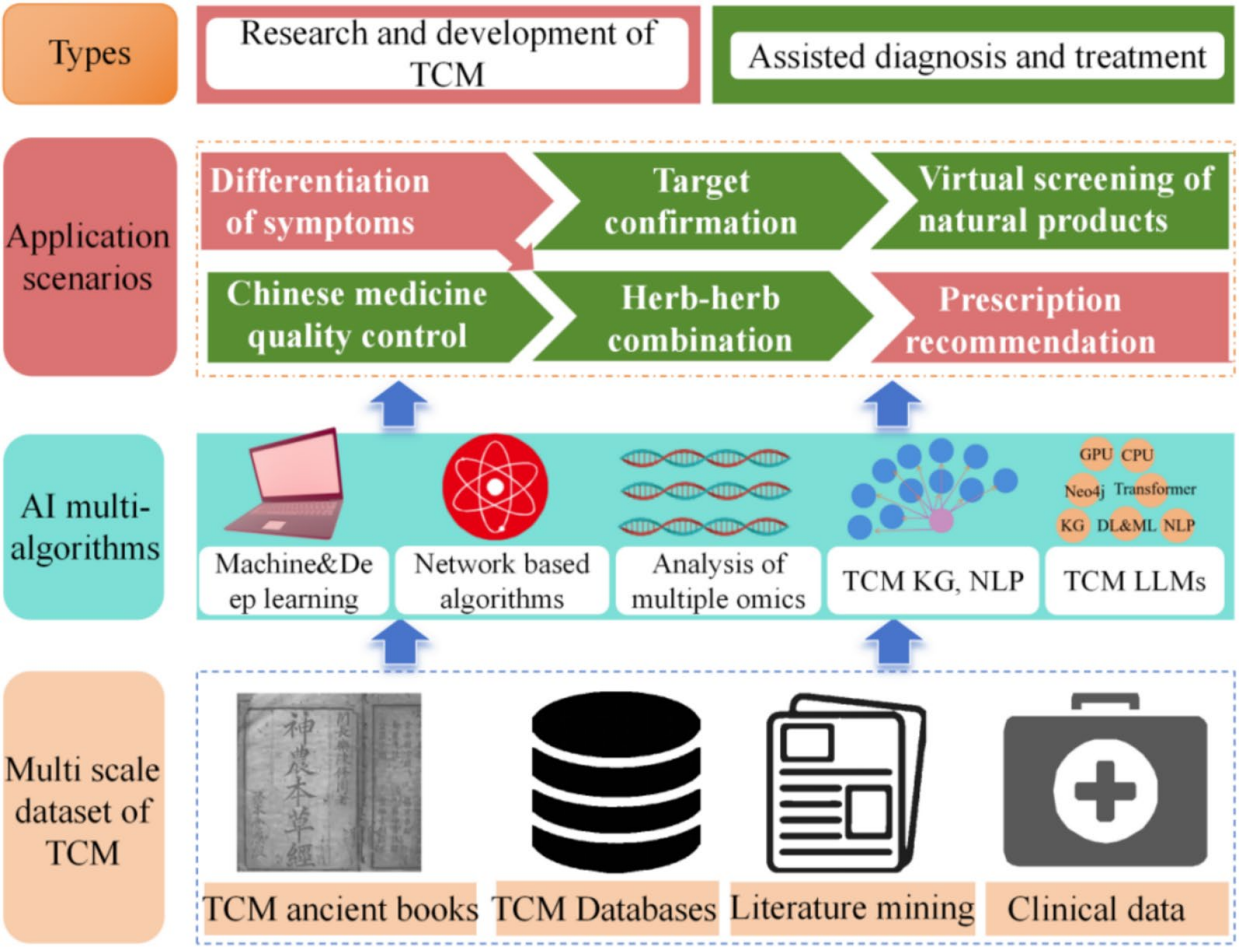
The general framework of AI in TCM (ML&DL primarily contribute to target identification, virtual screening, TCM quality control, and syndrome differentiation; Multi-omics approaches are effective for target identification and virtual screening; Network-based inference serves key roles in target identification, TCM quality control, herb-herb compatibility analysis, and prescription recommendation; KGs significantly support syndrome differentiation; LLMs can empower all the aforementioned TCM) R&D and clinical diagnosis/treatment scenarios, particularly when grounded in TCM multimodal data)

Currently, AI technologies have been applied to multiple areas of TCM research. This review provided a comprehensive summary of the various types of multi-scale knowledge bases in TCM, and introduced the diverse application scenarios of AI technology in empowering TCM research and practice (Fig. 1). Special emphasis is placed on the role of LLMs specifically tailored for TCM.

## Methods

**Fig. 2:**
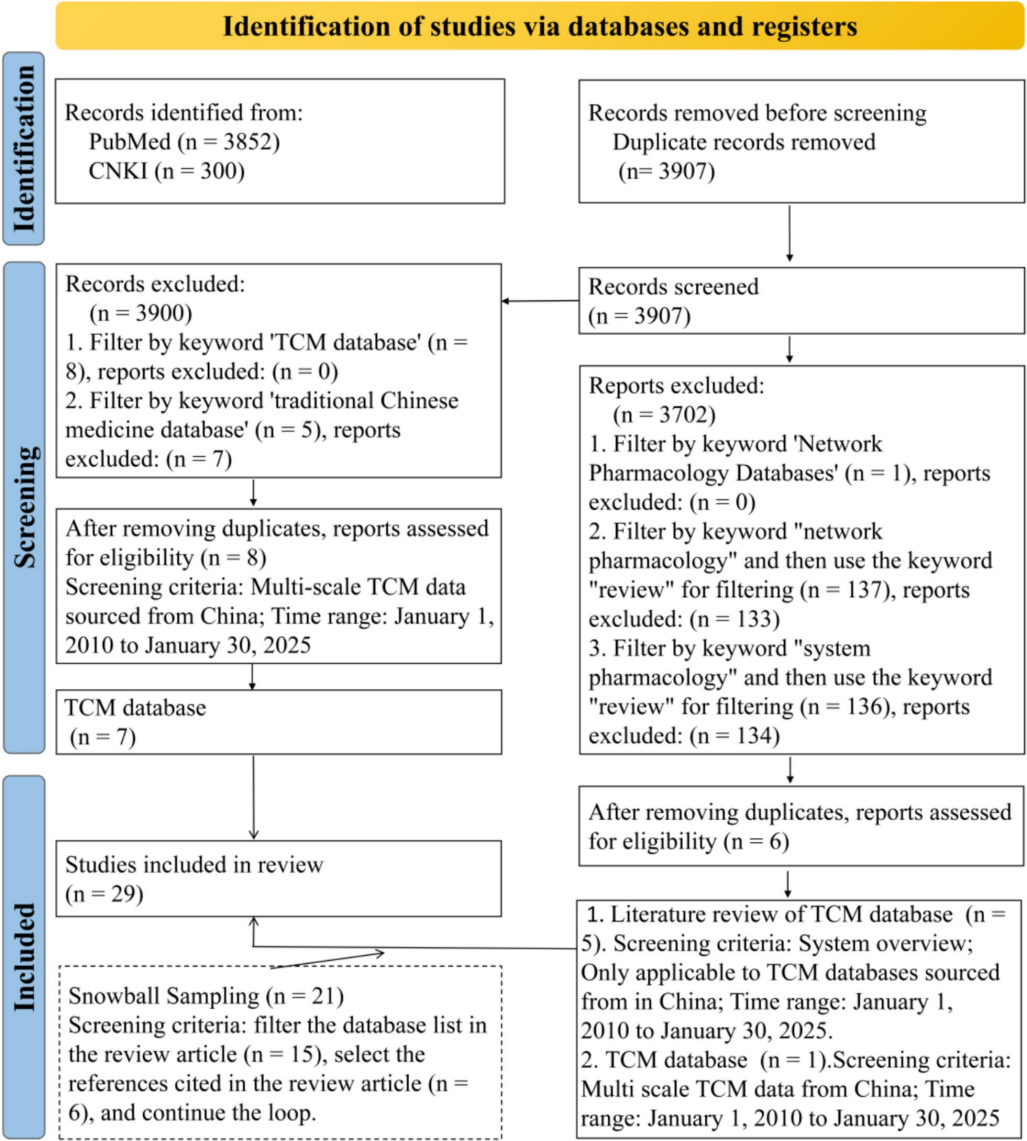
PRISMA flow diagram of the literature search

Our systematic review was conducted in accordance with the Preferred Reporting Items for Systematic Reviews and Meta-Analyses (PRISMA) guidelines. (1) Search Strategy: The databases PubMed and China National Knowledge Infrastructure [CNKI] were searched for publications focusing on the following 4 aspects: multiscale data resources for TCM, AI-driven TCM research and development, AI-assisted TCM diagnosis and treatment, and TCM generative pre-trained transformers, covering the period from 2010-01-01 to 2025-01-30. The terms [“Traditional Chinese Medicine” OR “herb” OR “Chinese herbal medicine” OR “traditional chinese patent medicines” OR “TCM” OR “traditional medicine” OR “herbal medicine” OR “plant” OR “herbal ingredients”) AND (“network pharmacology”) AND (databases)] were used to search for publications in order to include all the related researches; (2) Study Selection: Two independent reviewers screened titles/abstracts and full texts against eligibility criteria. Discrepancies were resolved through consensus or third-reviewer arbitration. The study selection process, including reasons for exclusion at each stage, is detailed in the PRISMA flow diagram (Fig. 2). (3) Data Extraction: In Microsoft Excel 2021, a standardized pilot form was used to extract data, including name of title, first author, publication year, citation, study design, discipline, and version of TCM databases used or mentioned in the article. Disagreements between the two investigators were discussed until consensus was reached; (4) Collating results: This study presented the findings of the included studies descriptively and categorized TCM databases into two primary sections: Multiscale data resources for TCM and multiple omics data resources for TCM sourced from China. According to Levac et al. [17], conducting systematic quality appraisal of included studies was deemed unnecessary, as the primary objective of a scoping review is to provide a broad overview of existing evidence.

## Multiscale data resources for TCM

**Fig. 3:**
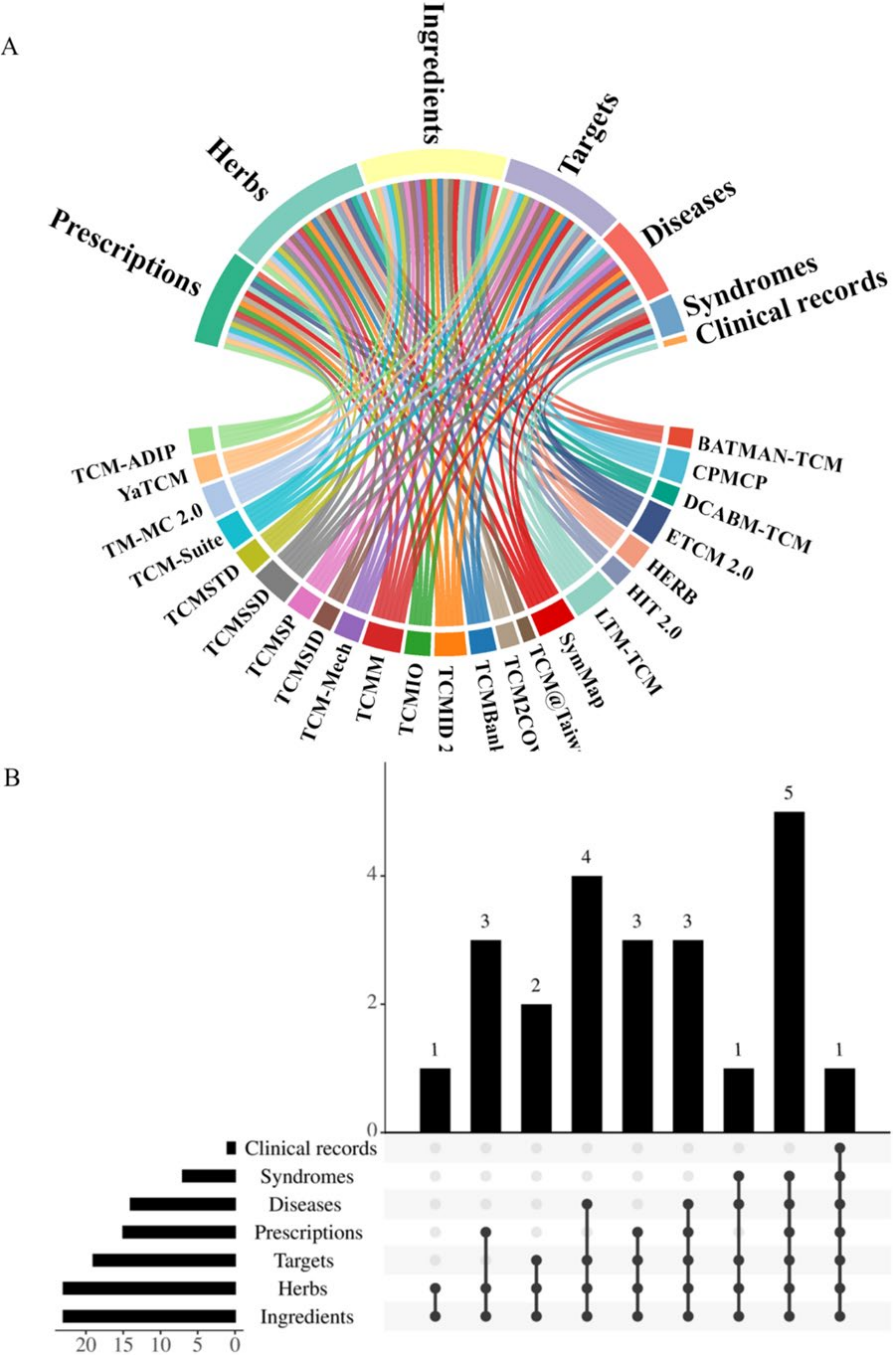
The multiscale data resources for TCM. **A** The circular diagram visually connects TCM databases to the types of data they cover, such as prescriptions, herbs, ingredients, targets, diseases, syndromes, and clinical records; **B** The bar chart on the right quantifies the number of databases that contain each type of data

Presently, the development of computational biology has enabled unprecedented depth and complexity in TCM data accumulation [3, 18]. TCM databases serve as a pivotal bridge, integrating traditional herbal formulas, individual herbs, and symptoms with modern medical concepts such as diseases, molecular targets, and biological pathways, enabling AI multiple algorithms analyses (Fig. 3). This review presents an overview of multiscale data resources for TCM, emphasizing their data sources and quality, data standardization methods, effects, data types, and research applications.

TCM databases usually adopt a multi-level standardized process and verification mechanism in data quality control to ensure the reliability, consistency, and academic value of the data [3]. Both the ETCM 2.0 and TCMSP databases prioritize sourcing information on TCMs, proprietary Chinese medicines, and marker ingredients from authoritative national-level references including the Pharmacopoeia of the People's Republic of China (2020 edition) and The Fourth National Survey on Chinese Materia Medica Resources (http://www.zyzypc.com.cn/), with each TCM entry documented using standardized Chinese names, Latin names, and source plant information [4, 19]; Ingredient data uniformly incorporates international identifiers such as PubChem CID and InChIKey sourced from specialized databases including PubChem, ChEMBL, and ChemSpider [18]; Ingredient targets and disease-associated genes are curated from DrugBank and BindingDB databases, while disease-gene relationships integrate data from MalaCards v5.0, Human Phenotype Ontology (HPO, 2018 Release), Online Mendelian Inheritance in Man (OMIM, April 2018 Release), and DisGeNET v5.0 [20]; Additionally, TCMSP predicts key compound properties including Lipinski’s Rule of Five parameters (molecular weight, AlogP, topological polar surface area, hydrogen bond donors/acceptors), drug-likeness (DL), oral bioavailability (OB), half-life (HL), and blood–brain barrier (BBB) permeability, while ETCM2.0 calculates drug-likeness using predictive models from the Bickerton Group [21–24].

TCM databases resources provided comprehensive annotations, syndrome standardization, modernization initiatives, and therapeutic innovations, such as LTM-TCM, ETCM 2.0, TCMSSD [4, 25, 26]. Additionally, (1) Disease-specific databases of TCM like TCM2COVID, TCMIO, and TCM-ADIP focused on anti-COVID-19 [27], immuno-oncology [28], and anti-Alzheimer’s disease [29], respectively. (2) It is worth noting that the databases DCABM-TCM [30], TM-MC 2.0 [31], TCMBank [32], HIT 2.0 [33], and HERB [34] leveraged literature evidence for mining herbal component-target interactions and pharmacotranscriptomics data. (3) The databases TCM-Mesh [35] and TCM-Suite [36] not only provided extensive data on the relationships between prescriptions, herbs, ingredients, targets, and diseases but also featured built-in platforms for automated NPM analysis. These platforms facilitate the intricate mechanism elucidation of TCM, screening of NPs, analysis of TCM combinations, prescription mining, and network visualization. (4) SymMap [37], CPMCP [38], TCMSTD 1.0 [39] and DCABM-TCM [30] databases focused on symptoms mapping, Chinese patent medicine mining, TCM system toxicology and constituents absorbed into the blood and metabolites of TCM, respectively. Meanwhile, these databases have been applied in various application scenarios, including the discovery of marker compounds (TM-MC 2.0), the identification of prototypes and metabolites experimentally detected (DCABM-TCM), the discovery of anti-cancer compounds and their targets (HIT 2.0, NPACT), and predictions of exclusions or interactions between Chinese medicines (TCMBank) (Table 1, Appendix 1).

**Table 1:** Multiscale data resources for TCM

Database	Prescriptions	Herbs	Ingredients	Targets	Diseases	Syndromes	Clinical records	Total	Effect	Refs.
LTM-TCM	48,126	9122	34,967	13,109	/	1928	41025	148277	Linking of TCM with modern medicine at molecular and phenotypic levels; drug discovery	[25]
ETCM 2.0	48,442	2079	38,298	1040	8045	319	/	98223	Mechanistic research; drug discovery; target identification; quality marker identification	[4]
TCMSSD	133518	8259	43413	17602	8073	624	/	211489	Syndrome standardization; syndrome prediction	[26]
TCMM	48043	8932	69816	76449	22365	1900	/	227505	Extensive TCM modernization knowledge; TCM modernization and therapeutic innovations	[42]
TM-MC 2.0	5075	635	34107	13992	27997	/	/	81806	Compound screening; drug discovery	[31]
TCM-Suite	6,692	7322	704,321	19,319	15,437	/	/	753091	TCM component identification; network pharmacology analysis	[36]
TCMID 2.0	15	778	18203	82	842	/	/	19920	TCM's modernization; exploring of underlying biological processes	[43]
SymMap	/	499	19595	4302	5235	1717	/	31348	Symptom mapping; phenotypic drug discovery	[37]
CPMCP	/	1557	26341	20,965	14086	2285	/	65234	Standardized TCM symptom; associations between TCM symptoms and MM symptoms	[38]
TCMIO	1493	618	126973	154	/	/	/	129238	Molecular mechanisms of TCM in modulating the cancer immune microenvironment	[28]

**Fig. 4:**
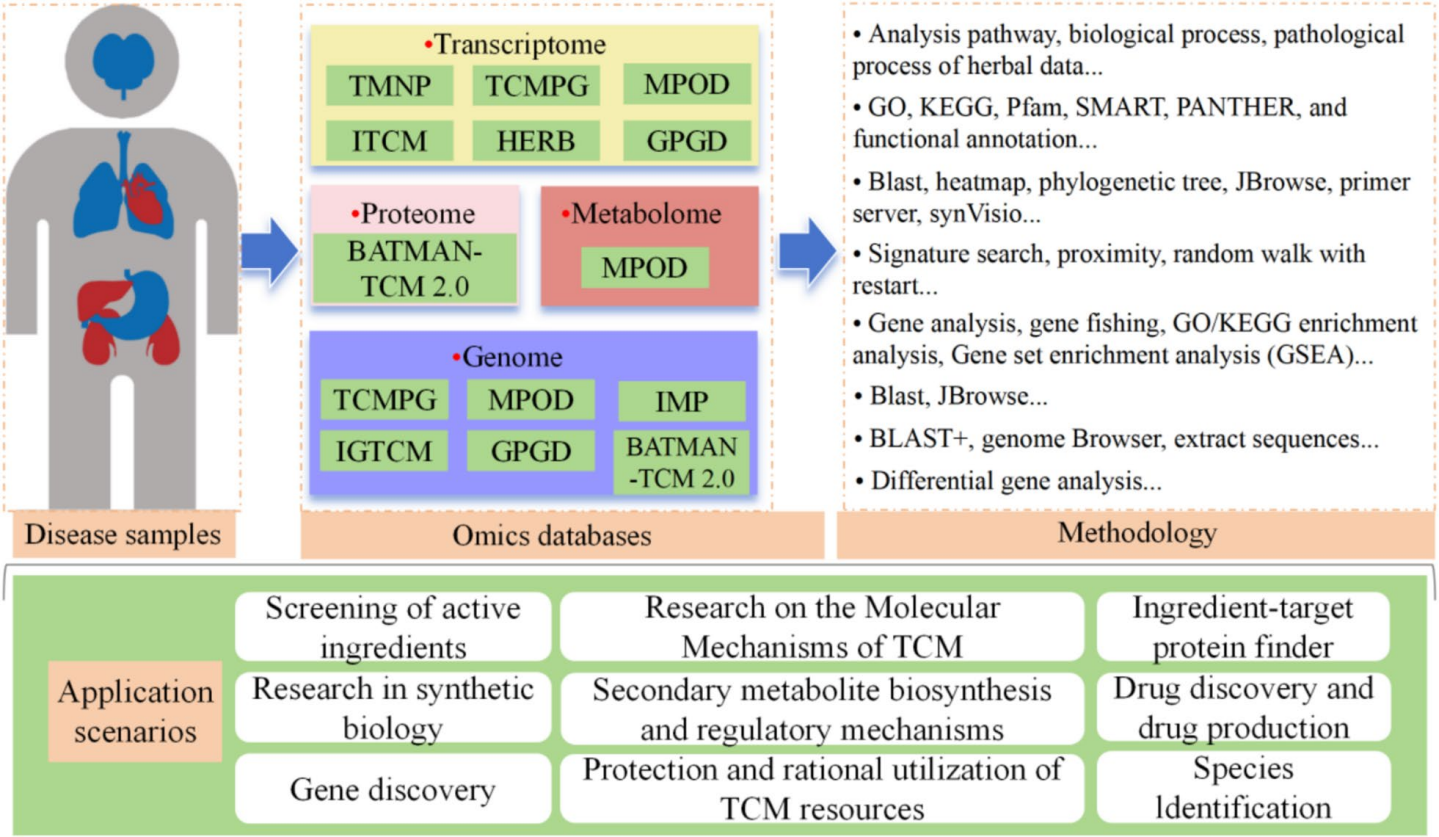
Summarized the integration of disease samples, omics databases, and methodologies to support research and application scenarios in TCM

TCM genomics databases played a crucial role in integrating and analyzing complex data from various aspects of plant biology, including genetics, genomics, transcriptomics, proteomics, and metabolomics. These databases provided comprehensive resources for researchers to explore the potential molecular mechanisms of TCM in treating diseases, as well as for analyzing and identifying NPs, target elucidation, controlling the quality of provides valuable data foundation (Table 2). For example, MPOD is designed to integrate multi-omics data from model plant species, providing a platform for comparative analysis across different plant genomes. It can facilitate research on gene function, metabolic pathways, and evolutionary relationships among plant species [40]. Tian et al. developed ITCM, the largest online pharmacotranscriptomics platform to date, for screening active ingredients [41]. Meanwhile, Fang et al. mapped 6,164 drug transcriptional signatures to the connectivity map (CMap) database, establishing connections between TCM/components and 2,837 modern drugs, guiding the rational development of modern drugs [34]. In addition, TCMPG, IMP, GPGD and IGTCM was specifically designed to integrate genomic and biochemical data related to plants used in TCM, applying to drug discovery and drug production, as well as research in synthetic biology, secondary metabolite biosynthesis, and regulatory mechanisms, Fig. 4.

**Table 2:** Multiple omics data resources for TCM

Database	Prescriptions	Herbs	Ingredients	Targets	Diseases	Genome	Transcriptome	Metabolome	Proteome	Effect	Refs.
ITCM	25857	5921	43426	18849	11180	/	√	/	/	Active ingredients screening	[41]
HERB	/	7263	49258	12933	28212	/	6164	/	/	Modern drug discovery	[34]
BATMAN-TCM 2.0	54832	8404	39171	/	/	√	√	/	√	Screen active TCM ingredients/herbs; known ingredient-target protein interaction	[44]
TMNP	/	/	/	/	/	√	/	/	/	NPM analysis	[45]
TCMPG	/	250	/	/	/	195	/	/	/	Comparative and functional genomic analysis	[46]
IGTCM	/	83	1033	3610350	/	83	/	/	/	Drug discovery	[47]
IMP	/	1024		39,581,852		84	/	/	/	Drug discovery and drug production	[48]
MPOD	/		85	/	/	6	28	5	/	Research in synthetic biology	[40]
GPGD	/	> 1000	/	/	/	792	9682	/	/	Secondary metabolite biosynthesis and regulatory mechanisms	[49]

In summary, this integration facilitated a deeper understanding of the mechanisms underlying TCM therapies and enhances their application in TCM/NPs discovery and auxiliary diagnosis and treatment.

## The integration of AI and multi-scale data in the research and development of TCM

### NPs target discovery

By identifying new drug targets, we can not only deepen our understanding of the complex mechanisms by which TCM formulas and their active ingredients treat diseases but also promote the discovery of novel NPs [50]. The main methodologies for identifying targets of NPs include ML/DL-based predictions, ligand similarity screening, pharmacophore matching, shape similarity analyses, gene or cell expression profiling, network-based inference, clustered regularly interspaced short palindromic repeats (CRISPR), and single-cell omics, etc. (Table 3 and Appendix 2).

**Table 3:** AI-driven platform and database for natural product target prediction

Systems	Methods	Advantage	Limitation	Webserver	Refs.
TCMSP	SysDT model	Multi algorithm fusion prediction (RF, SVM), Cross scale data integration (chemical, genomic, and pharmacological information) [58]	Relying on the coverage of existing knowledge base	http://sm.nwsuaf.edu.cn/lsp/tcmsp.php	[19]
SuperPred 3.0	Structural similarity of ECFP molecular fingerprint	Revealing the binding mechanism, Discovering a brand new target, high specificity	Insufficient flexibility in handling, deviation of scoring function	https://prediction.charite.de/subpages/target_prediction.php	[52]
SwissTargetPrediction	The similarity of 2D and 3D structures			http://swisstargetprediction.ch/	[51]
LigandScout	Reversed pharmacophore matching	Breaking through the dilemma of"orphan ligands", high mechanism interpretability, multi target synergistic effect, high resource efficiency	Dependent on the quality of the target pharmacophore library, natural product specificity error	http://www.inteligand.com/pharmdb/	[55]
PharmMapper				http://lilab.ecust.edu.cn/pharmmapper/	[56]
TCMIO/bSDTNBI	Balanced substructure-drug-target network-based inference	Prediction of synergistic targets of multiple components in TCM, high noise resistance and data compensation capability, balance the importance weight of target points	Subjectivity of substructure definition, insufficient dynamic control modeling	http://lmmd.ecust.edu.cn/netinfer	[28, 75]
CMap	Drug-induced gene expression profile	Directly associated phenotype, breaking through the limitations of the knowledge base, revealing dose dependence	Masking of cellular heterogeneity, complexity of data interpretation	http://portals.broadinstitute.org/cmap/	[76]
DeepPurpose	DL	Breaking through the single representation of structure, high precision prediction, data utilization efficiency	Data dependency and bias amplification, interpretable black hole	https://github.com/kexinhuang12345/DeepPurpose	[60]
DeepDTA				https://github.com/hkmztrk/DeepDTA	[61]
ReduMixDTI	Feature redundancy reduction and interpretable attention mechanism	Reduction of feature redundancy, mechanism transparency, leap in computational efficiency	Information loss risk, attention misleading trap	https://github.com/mql430/ReduMixDTI	[64]
CRISPR	Clustered regularly interspaced short palindromic repeats	The gold standard for causality verification, whole genome unbiased screening, compatibility of in *vitro* and in *vivo* models	Channel compensation problem, high technical threshold and cost		[77]

SysDT: Systems dynamics and therapeutics; RF: Random forest; SVM: Support vector machine; CRISPR: Clustered regularly interspaced short palindromic repeats

#### Target predicted based on ligand similarity

**Fig. 5:**
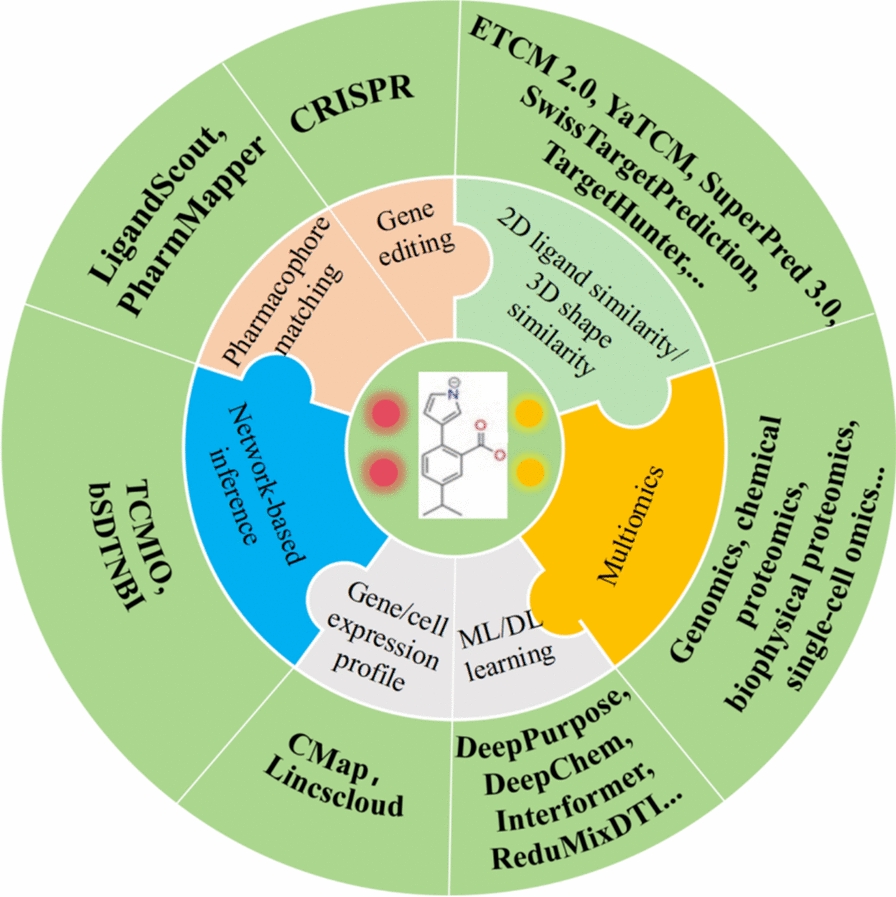
Natural product target prediction methods

Ligand-based screening methods played a crucial role in modern drug discovery processes, especially in the field of NP target prediction. As shown in Fig. 5, numerous databases and predictive tools have been developed, offering rich resources for identifying potential therapeutic targets and drug candidates. For example, SwissTargetPrediction used both 2D and 3D structural similarities to predict the potential targets of compounds while SuperPred 3.0 utilized structural similarity based on extended-connectivity fingerprint (ECFP) to predict NP-target interactions [51, 52]. Liu et al. used SwissTargetPrediction to predict the targets of Agrimonolide and ultimately discovered that Agrimonolide inhibits cancer progression by targeting SCD1 [53]. Dao et al. utilized the SuperPred website to predict and verify that Gramine improves sepsis-induced myocardial dysfunction by binding to NF-κB p105 [54]. Additionally, tools like LigandScout and PharmMapper employed inverse pharmacophore matching techniques [55, 56]. These methods not only take into account the shape and chemical characteristics of small molecules but also integrate their ability to interact with specific proteins, leading to more accurate and reliable predictions. He et al. utilized both SwissTargetPrediction and PharmMapper methods to identify NLRP3 as the target responsible for the neuroprotective effects of Asiaticoside, providing new insights into the mechanisms underlying its therapeutic effects on neurological conditions [57].

#### Target predicted based on ML/DL

With the development of ML/DL algorithms, ligand-based screening methods are becoming increasingly intelligent in drug discovery and target prediction. For example, the TCMSP database employs systems dynamics and therapeutics (SysDT) model, achieving 82.83% concordance, 81.33% sensitivity, and 93.62% specificity in predicting NPs-target interactions [19, 58]. Yang et al. employed the SysDT model to predict the targets of NPs and, through subsequent enrichment analysis, identified critical pathways such as nuclear factor kappa-B (NF-κB) and Tumor necrosis factor (TNF) signaling, along with the phosphatidylinositol 3-kinase (PI3K)-the serine/threonine kinase (Akt) and C-type lectin receptor pathways. Their computational predictions aligned closely with experimental observations [59].

In addition, DL framework specifically designed for NP-target interaction prediction, such as DeepPurpose and DeepDTA. It supported various data input formats and integrated different DL models, allowing users to flexibly choose the optimal configuration based on their specific needs [60, 61]. For example, by utilizing DeepPurpose in conjunction with molecular docking studies, Yan et al. identified MAPK14 as one of the potential targets of ginkgolides [62]. Additionally, Interformer introduced a graph-transformer architecture, which is particularly suited for handling complex molecular structure information, thereby improving the understanding of interactions between NPs and targets [63]. ReduMixDTI enhanced the interpretability and performance of NP-target interaction predictions through feature redundancy reduction and interpretable attention mechanisms [64].

#### Target predicted based on multi-omics

In recent years, single-cell multiomics atlas have enabled the isolation of individual cells from samples, followed by genomic sequencing, transcriptomic sequencing, proteomic analysis, and metabolomic analysis. This approach offers richer information for the discovery of NPs targets and effect pathways (Table 4). The concept of multi-omics also aligns well with the “*holistic view*” and “*systems view*” in TCM [65–67]. For example, Deng et al. discovered through single-cell multi-omics that cycloastragenol targets cathepsin B to enhance antitumor immunity of CD8 T cells by inhibiting major histocompatibility complex 1 (MHC-I) degradation [68]. Gao et al. identified exportin-2 (CSE1L) as a potential target for the marine natural product Naamidine J in the treatment of acute lung injury through chemoproteomics [69]. Piaz et al. used the drug affinity responsive target stability (DARTS) method to identify Laurifolioside as a new modulator of the clathrin heavy chain [70].

**Table 4:** Multi-omics based method for target prediction of NPs

Omics	Type	Effects	Advantage	Limitation	Refs.
Single-cell omics		Simultaneous analysis of information at multiple biomolecular levels at the single cell level	Identify rare cell subpopulations and accurately locate potential targets	Challenge of low abundance target detection	[66]
Genomics	CMAP	Drug-induced gene expression data of cell lines	Genome-wide association study, rare disease target mining	Difficulties in interpreting non coding areas, redundancy of multi gene disease targets	[78]
Chemical proteomics	CCCP	Active small molecule probes detect intracellular proteomic changes for potential drugs	Understanding of specific natural products structure–activity relationships	Only suitable for identification of protein targets with a high abundance and affinity to their corresponding immobilized small molecules	[79]
	ABPP	ABPs are used to selectively label and enrich proteins that are in an active state	Directly profile active ligand and functional sites in complex proteomes	Limited to binding only a few amino acid residues, including cysteine and lysine	[80]
Biophysical proteomics	DARTS	The sensitivity of proteins to protease digestion in the presence or absence of drugs was compared	Reveal natural product target protein without the need for chemical modification and immobilization	Inability to identify minute low-abundance proteins	[81]
	SPROX	To identify the interactions between small molecule drugs and proteins and their effects on protein stability	Quantify natural product binding affinities to target proteins	Not suitable for detecting peptides that do not contain methionine	[82]
	CETSA	Drug targets are identified and validated by monitoring changes in the thermal stability of proteins within cells	Estimate drug binding targets in intact cells, cell lysates, and tissue samples	Suitable for validating known protein targets but is limited in its ability to discover unexpected or novel targets	[83]
	TPP	Interactions between small molecules and protein targets are detected by monitoring the sensitivity of proteins to protease digestion at different temperatures	Evaluate ligand-target engagement in vitro, in situ, or in vivo	Applicable only to soluble protein components and insensitive to low-abundance proteins	[84]

CMAP: Connectivity Map; CCCP: Compound-centric chemical proteomics; ABPP: Activity-based protein profiling; DARTS: Drug affinity responsive target stability; SPROX: Stability of proteins from rates of oxidation; CETSA: Cellular thermal shift assay; TPP: Thermal proteome profiling; ABPs: Activity-based probes

#### Target predicted network-based inference

In modern drug discovery, network-based methods offer a systematic and comprehensive strategy for predicting the targets of NPs. This approach integrated TCM data and computational models to construct complex molecular interaction networks, thereby identifying potential NP targets and elucidating how NPs exert their biological effects within cells [71, 72]. The TCMIO database employs the bSDTNBI approach to predict targets for 120,000 NPs, aiming to expand the identification of new targets and indications for these compounds. Additionally, TCMIO has predicted that curcumin targets signal transducer and activator of transcription 3 (STAT3). Experimental validation confirmed that curcumin exhibited significant inhibitory activity on IL-6-induced STAT3 activation, with an IC50 value of 1.6 µM [28]. Wu et al. developed a network-based inference method called NetInfer, and the results from predicting and experimentally validating cardiovascular diseases and their potential molecular mechanisms were consistent [73]. Zhou et al. employed bSDTNBI-FCFP_4 molecular docking method to identify the target proteins of sarsasapogenin derivatives (SDS). Through subsequent in vitro validation, they discovered that uclear factor erythroid 2-related factor 2 (Nrf2) serves as a crucial target for the anti-Alzheimer's disease (AD) effects of these SDS [74].

While ligand similarity-based, ML/DL, multi-omics, and network inference-based methods demonstrated significant capabilities and broad application prospects in NP target prediction, it is crucial to emphasized that each prediction approach possesses distinct advantages alongside inherent limitations. For instance, ligand similarity methods rely heavily on the comprehensiveness of known ligand information, ML/DL model performance is contingent upon the quality and scale of training data, multi-omics techniques involve high costs and complex data analysis, and the accuracy of network inference is intrinsically linked to the completeness of the constructed network. Therefore, in practical applications, researchers must judiciously select and integrate the most suitable prediction methods, or even necessitate experimental validation, based on the specific requirements of the research objective, available data resources, and the characteristics of the NP under investigation, to obtain reliable and valuable target prediction outcomes (Table 3 and Appendix 2). In short, strategies for NPs target discovery empowered by AI not only serve as a critical bridge connecting AI and basic research but also act as a driving force for continuous innovation in TCM.

### Natural products virtual screening

In recent years, scientists have shown significantly increased interest in NPs as a source of novel drugs, particularly in the potential of TCM components for drug development from 1981 to 2019 [85, 86]. For example, artemisinin, widely acknowledged as a gift from TCM to the global community, has rescued the lives of countless individuals suffering from malaria [87]. Traditional high-throughput screening is time-consuming and resource-intensive. The emergence of AI has accelerated the pace and increased the success rate of novel drug discovery from NPs.

#### SBVS

Recently, the advent of AlphaFold3 has revolutionized the field of structural biology by accelerating the accurate prediction and discovery of 3D protein structures. By providing highly detailed and reliable structural models, AlphaFold enabled researchers to identify potential drug targets more efficiently and design novel therapeutics with greater precision. This breakthrough technology significantly expedited the process of drug discovery, especially structure-based virtual screening (SBVS) [88, 89]. Adeena Tahir et al. employed an integrative approach combining a β-N-acetyl-D-hexosaminidase model generated using AlphaFold, alongside molecular docking and molecular dynamics (MD) simulations. This comprehensive strategy led to the identification of 5 compounds that exhibit significant binding affinity to the OfHex1 thioredoxin-related transmembrane active site [90]. In addition, VS tools such as MTiOpenScreen [91], DockThor-VS [92] and e-LEA3D [93] also played an important role in the discovery of NPs with high theoretical accuracy, computational efficiency, and low experimental cost, which are characteristic features of scaffold hopping. However, it cannot achieve large-scale batch screening (Appendix 3). Fortunately, structure-based accelerated VS platform has been developed, which can screen compounds more efficiently, such as RosettaVS [94] and Roboticized AI [95].

#### LBVS

Furthermore, ligand-Based virtual screening (LBVS) includes pharmacophore modeling, substructure and fragment search, quantitative structure–activity relationship (QSAR), shape and pharmacophore similarity search, fingerprint matching, DL/ML, etc. Meanwhile, scientists have developed a variety of databases based on LBVS theory that are easy for researchers to use and speed up drug development, such as ChemDes, ChemMine Tools and BRUSELAS (Table 5, Appendix 3). However, current LBVS methods rarely consider the 3-D conformational space of molecules, often neglecting the dynamic properties and conformational flexibility of compounds. The GeminiMol model addressed this limitation by incorporating the conformational space profile of compounds into molecular characterization learning. This approach extracts features that represent the complex interactions between molecular structure and conformational space, thereby promoting the formation of a new paradigm in LBVS [96].

**Table 5:** AI-based VS platform for NPs

Database	Classic	Methods	Advantage	Limitation	Refs.
MTiOpenScreen	SBVS	AutoDock/AutoDock vina	High theoretical accuracy, efficiency and low cost, scaffold hopping, suitable for targets without ligand information, evaluate the stability of molecular dynamic simulation	Highly dependent on the quality of target structure, reliability of scoring function, difficulties in modeling solvation and entropy effects, incomplete coverage of chemical space, false positive and false negative risks	[106]
DockThor-VS		Multiple-solution genetic algorithm and the MMFF94S molecular force field			[107]
e-LEA3D		Genetic algorithm			[108]
RosettaVS		RosettaGenFF-VS	Simulate the motion of its flexible side chains and finite main chain	Incomplete coverage of chemical space	[94]
Roboticized AI		Microfluidic photocatalytic synthesis	Fully automated, high-throughput photocatalytic reaction, characterization, and screening system	Further improved to meet the needs of different types of photocatalytic reactions	[95]
ChemDes	LBVS	Computing molecular descriptors and fingerprints	No target structure required, high computational efficiency, small-sample friendly, circumvents the protein flexibility challenge, and integrates multi-dimensional ligand information	Highly dependent on the quality of known activity data, limited scaffold innovation, poor cross-target generalization capability, and fails to deliver ligand-target interaction details	[109]
ChemMine Tools		Fingerprint and embedding/indexing algorithms			[110]
BRUSELAS		3D shape and pharmacophore searches	Not dependent on specific chemical structures/skeletons, suitable for situations where the target structure is unknown	Ignore chemical characteristics, highly sensitive to conformation	[111]
DeepScreening		Classification and regression models	Quickly construct DL models, de novo generates a chemical library and use those models for VS	Modeling data relies on public databases	[112]
OCHEM/MLViS		QSAR	Quantitative structure–activity relationship, used when the target structure is unknown, suitable for large-scale screening	Highly dependent on the quality of training data, the contradiction between descriptor selection and model complexity	[113, 114]
GeminiMol		Capturing the complicated interplay between the molecular structure and the conformational space	Enable training reliable molecular representation models without including experimental molecular properties	Unable to screen compounds with special structures such as covalent compounds and metal ion coordination compounds	[96]
ITCM	VS based on multiple omics	Transcriptomics	Multidimensional target identification, enhancing clinical relevance, circumventing structural dependencies, accelerating hit compound discovery	High complexity of data integration, challenges in experimental validation of multi-omics mechanisms, scarcity of omics data for rare diseases, inadequate capture of dynamic biological processes	[41]
HERB		Transcriptomics			[34]
BATMAN-TCM 2.0		Transcriptomics, genomics, proteomics			[44]
TCMSP	VS based ADME filtering	MW, HL, OB, DL, BBB pharmacokinetic properties…	Eliminating molecules with poor drug-likeness, significantly reducing costs, computational efficiency, enhanced clinical translation potential	Oversimplification of physiological complexity, highly variable reliability of prediction models, lack of in vivo dynamic data, and neglect of transporter interactions	[19]
SwissADME		Physicochemical properties, pharmacokinetics, drug-likeness and medicinal chemistry friendliness, among which in-house proficient methods			[115]
ADMETlab 3.0		21 physicochemical properties, 19 medicinal chemistry properties, 34 ADME endpoints, 36 toxicity endpoints, and 8 toxicophore rules			[116]
BATCHIE	Combination drug screens	Bayesian	Take any Bayesian model and design optimal batches with respect to that model	Limited by the data on which they are trained	[105]

ADMET: Absorption, Distribution, Metabolism, Excretion, Toxicity; MW: Molecular weight; HL:half-life; OB:Oral bioavailability; DL: drug-like; BBB: Blood–brain barrier

#### VS based on multiple omics/ADME filtering

**Fig. 6:**
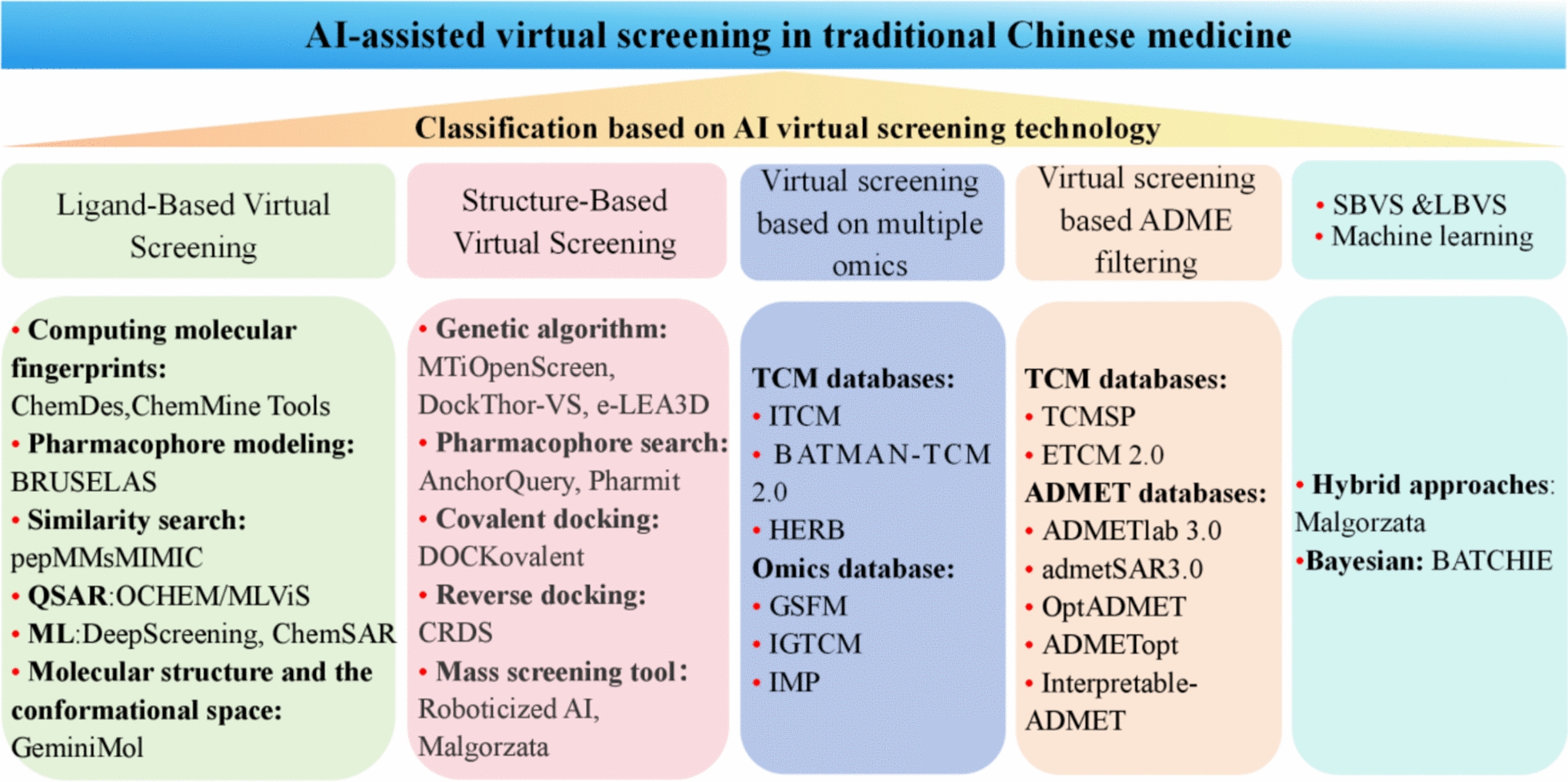
Application of AI in virtual screening of NPs

**Fig. 7:**
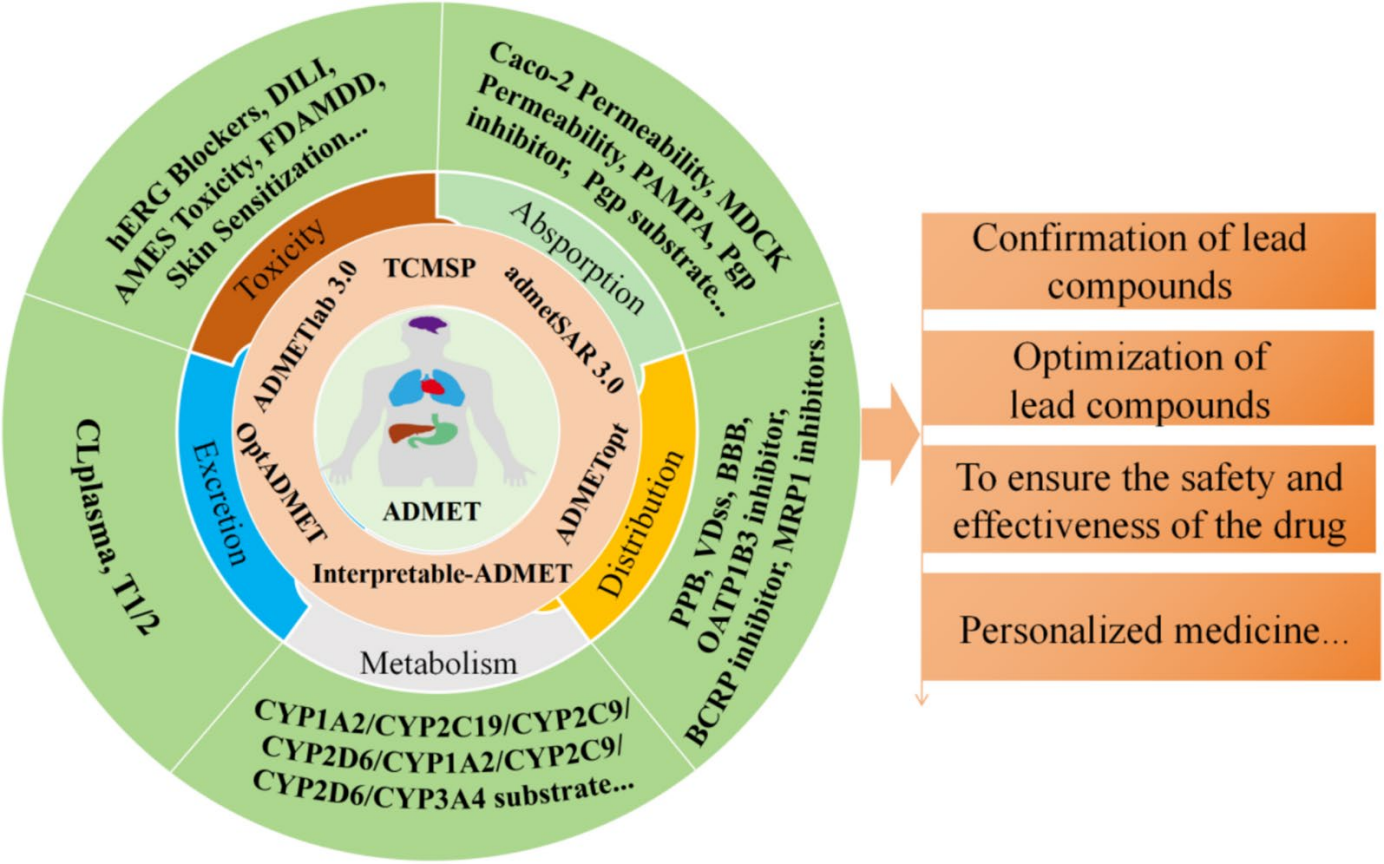
Natural product discovery based on ADMET

TCM databases such as ETCM 2.0, BATMAN-TCM 2.0, and HERB provided a highly valuable data foundation for drug screening, such as leveraging omics data and ADMET-based pharmacokinetic properties (Fig. 6). For example, Duan and colleagues developed a transcriptotype-driven screening system utilizing the HERB and ITCM databases. Using this innovative approach, they identified apigenin as a promising therapeutic candidate for cholestatic liver fibrosis by inhibiting the downstream inflammatory responses induced by type-I interferon [97]. The TCMSP database performs VS of NPs by incorporating parameters related to absorption, distribution, metabolism, and excretion (ADME). This integrated approach ensured that only compounds with favorable pharmacokinetic properties are selected for further investigation [19]. Shen et al. integrated data from the TCMSP and TCM @Taiwan databases, applying ADME/T properties filtering to identify active components within Xiao-Xu-Ming Decoction for the treatment of AD. Through this comprehensive screening process, they successfully identified Fangchinoline and Dauricine as potential therapeutic compounds [98]. Huang et al. utilized the TCMSP and PubChem databases to identify the main components of Sanhua Decoction and employed the SwissADME online system for ADMET screening. Their findings revealed potential compounds in Sanhua Decoction that could treat ischemic stroke by targeting multiple signaling pathways [99]. Yi et al. discovered novel diazine derivatives as potential xanthine oxidase inhibitors through a combination of quantitative structure–activity relationship (QSAR), molecular docking, and ADMET properties screening using ADMETLAB 2.0 and SwissADME [100]. Additionally, other ADMET tools include admetSAR 3.0, OptADMET, ADMETopt, and Interpretable-ADME (Table 5, Appendix 3). These tools are applied in the validation and optimization of lead compounds as well as in drug safety assessments [101, 102] (Fig. 7). Simultaneously, it should be noted that ADMET-based screening exhibits limitations including oversimplification of physiological complexity, considerable variability in prediction model reliability, and absence of in vivo dynamic data[103]. While omics-based screening exhibits over-reliance on existing datasets [97].

Notably, the scientists have been able to apply a combination of LBVS and SBVS-based molecular modeling methods to VS [104]. Recently, Tosh and colleagues developed BATCHIE, a novel approach to combinatorial drug therapy, by employing Bayesian active learning, which involves dynamically refining the experimental design in response to accumulating data. This approach represented a groundbreaking solution for the screening of combination drugs, significantly enhancing the efficiency and effectiveness of identifying optimal drug combinations [105].

### Chinese medicine quality control

Compared to Western pharmaceuticals, TCM are characterized by their “multi-components, multi-targets” synergistic effects, which constitute a significant feature of these herbs. Establishing a rational, accurate, and theory-congruent quality evaluation system for TCM is imperative, especially in line with the characteristics of TCM [117, 118].

#### Prediction of medicinal properties and efficacy of TCM

Firstly, the therapeutic effects of herbal medicines are intimately linked to their inherent properties, which encompass the *four qi* (warm, hot, cold, cool), *five tastes* (pungent, sweet, sour, bitter, salty), *meridian tropism* (the meridians through which the herb acts), and toxicity. For example, Gao et al. performed pre-training on 31,114 unlabeled TCM formulas by evaluating their *four qi*, *five tastes*, *channel tropism*, and *toxicity*. Subsequently, they carried out two in-domain classification tasks for prediction and fine-tuning, ultimately aiming to determine the efficacy of these TCM formulas [119]. In addition, Wang et al. identified the top compound features important for *meridian* prediction among 646 TCM based on fingerprints and ADME properties while Yeh et al. utilized a cost-sensitive GCN network model to analyze both local and global topological features of chemical compounds, thereby uncovering the associations between TCM and their respective *meridians* [120, 121]. Additionally, several specialized databases of molecular tastants have employed ML and DL techniques to build models that correlate molecular structures with taste characteristics. These models enable the prediction of both the chemical structures of novel molecules and their likely taste categories, offering a modern scientific framework for interpreting the traditional TCM theory of medicinal properties and flavors (Table 6).

**Table 6:** AI-enabled quality control in TCM

Systems	Methods/models	Application	Refs.
	Genome, transcriptome and methyl group	TCM flowering time prediction	[124]
TCM2Vec	FMh2v	Formula efficacy prediction	[119]
	SVM, DT, RF and kNN	Meridian predicting	[120]
	GCN network		[121]
ChemTastesDB	Unsupervised ML approaches	Molecular tastants predicting	[132]
	Shapley additive explanations		[133]
NB-TCM-CHM	MobileNetV2	TCM graphics classification	[134]
	MLP regression model	Main components predicting	[135]
TCM-TextCNN	TF-IWF algorithm	TCM processing and decocting time	[126]
	Metabolomics		[125]
	Omics; systems pharmacology	TCM Q-marker predicting	[130]
	ADME studies; systems biology		[129]
	NPM, molecular docking, and MD simulations		[131]

FMh2v: Cross-feature-based unsupervised pretraining model; TF-IWF: Term frequency-inverse word frequency; MLP: Multilayer perceptron; MD: Molecular dynamics simulation; RMSD: root mean square deviation; rg: gyration

#### Harvesting and processing of TCM

**Fig. 8:**
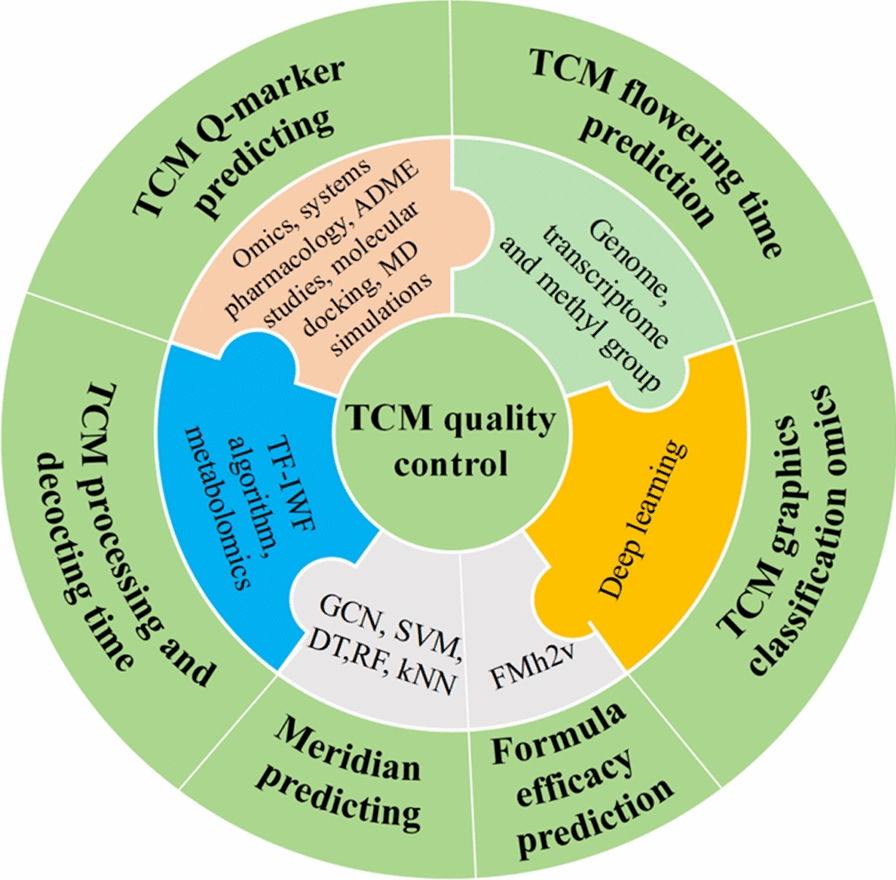
Application of AI in TCM quality control

In addition to the theory of TCM efficacy, the quality standards of TCM are also influenced by the harvesting practices, processing methods, authentication of genuineness, and the identification of quality markers (Q-marker) for the herbs [122]. AI and bioinformatics techniques have offered novel strategies and profound insights for enhancing the quality control of TCM (Fig. 8) [123]. For example, Wang et al. integrated genomic, transcriptomic, and methylomic data from *Arabidopsis thaliana* to successfully develop predictive models for six key traits. Notably, the flowering time models derived from different datasets identified distinct benchmark genes, underscoring the feasibility and effectiveness of integrating multi-omics data to elucidate the molecular mechanisms of complex traits [124]. Studies have shown that different processing methods and decoction processes significantly impact the pharmacological components of TCM. Chen et al. investigated the material changes in *Panax ginseng* and *Panax quinquefolius* under various processing methods using metabolomics and other techniques. They found that decoction times exceeding two hours had the most pronounced effects on the compounds within the ginseng [125]. Meanwhile, Li et al. proposed TCM-TextCNN, an improved text classification method using NLP that integrated multi-dimensional herb features and an enhanced TF-IWF algorithm to accurately predict TCM decoction durations [126].

#### Screening of TCM Q-marker

Finally, TCM is a multi-component complex system, and the quantitative evaluation of its active ingredients or effective fractions is crucial for establishing a quality control system for TCM [127, 128]. Traditional chemical analysis methods, such as high-performance liquid chromatography (HPLC), gas chromatography (GC), and mass spectrometry (MS), played crucial roles in the quality control of TCM. However, these methods also have several limitations, including complex sample preparation, high equipment costs and lengthy analysis times, especially in simultaneously analyzing multiple components. Presently, advanced methodologies including DL, NPM, multi-omics approaches, and virtual screening are significantly accelerating the discovery and identification of Q-marker in TCM. For instance, Wang et al. identified notopterol, isoliquiritin, imperatorin, cimifugin, and glycyrrhizic acid as Q-markers for the treatment of rheumatoid arthritis from Qianghuo Shengshi decoction through ADME studies, systems biology, and experimental verification methods [129]. Gao et al. combined metabolomics and systems pharmacology to screen for six Q-markers from carbonized TCM, which were then validated using an in vivo high-throughput screening model [130]. In addition, VS, molecular docking, and MD simulations also play a significant role in the discovery of Q-markers [131].

## The integration of AI and multimodal data of TCM for disease diagnosis

**Fig. 9:**
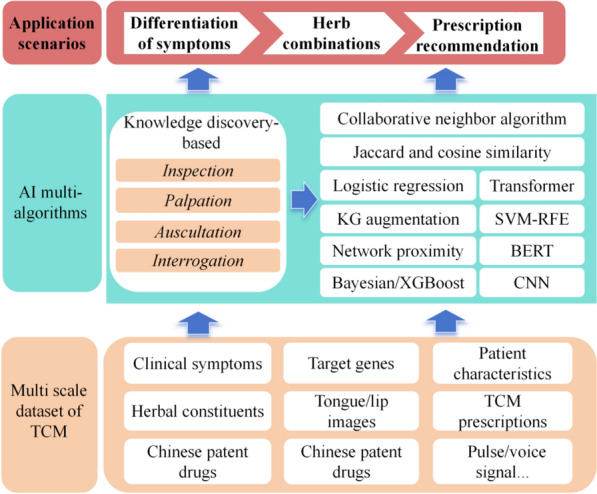
Application of AI in symptom differentiation and prescription recommendation

TCM diagnostic methods mainly consist of four diagnostic techniques: *observation*, *olfaction*, *inquiry*, and *palpation *[15, 136]. These methods involved assessing the patient's external characteristics such as facial complexion, tongue appearance, body posture, voice features and respiratory odors and evaluating changes in the functions of internal organs to diagnose conditions. Finally, according to the symptoms, the corresponding Chinese medicine prescriptions were prescribed. However, traditional diagnostics heavily relied on the subjective judgment and personal experience of doctors, leading to inconsistencies or misdiagnoses due to individual differences [137]. With the advancement of AI technologies, particularly in KG augmentation, transformer, and CNN, AI could achieve diagnostic results that match or even surpass those of clinical experts by learning from large datasets of TCM knowledge. This approach reduced biases arising from personal experience and subjective judgments, enabling more precise medication prescriptions tailored to specific symptoms. Consequently, it assists doctors in making more accurate therapeutic decisions, thereby providing strong support for personalized medicine [1, 138] (Fig. 9).

### Differentiation of symptoms

The differentiation of symptoms is an important method in TCM for treating diseases. In recent decades, modern medicine has demonstrated that elucidating the relationship between symptoms and diseases is crucial for discovering disease etiologies and developing new drugs. For example, Zhou et al. developed a human symptoms-disease network to clarify the connection between the molecular origins of diseases and their corresponding phenotypes in the post-genomic era, providing insights that are crucial for understanding disease mechanisms and improving diagnostics and therapies [139].

#### Knowledge discovery-based differentiation of symptoms

Mining TCM knowledge is the primary method for AI to perform symptom differentiation. Knowledge discovery could utilize TCM databases, clinical electronic records, ancient books, and pharmacological literature to reveal the relationships between diseases and patterns [140, 141]. Yin et al. constructed a tinnitus KG by integrating electronic medical records (EMRs) data from 1,267 patients with TCM clinical knowledge. They utilized a collaborative neighbor algorithm for the differentiation of tinnitus symptoms [142]. TCMSSD leveraged DL and KG approaches, including the BM25 algorithm, to accurately predict syndromes. These AI algorithms and methodologies have enabled researchers to extract meaningful insights from complex multi-dimensional data, facilitating more precise diagnoses, personalized treatment plans, and innovative TCM/NPs development [26]. Ye et al. created a KG based on TCM characteristics, integrating medical text with KG entity representation through a method called Fused Medical Text and KG Entity Representation (F-MT-KER) to obtain prediction results. Finally, they utilized the BERT model for vectorization to better simulate the TCM syndrome differentiation of complex cases [143].

#### Symptom dialectics based on the four diagnostic parameters

Identification of tongue shape and texture features is an important aspect of symptom differentiation in the visual *inspection* (望) of TCM. Jiang et al. categorized tongue images into seven classes based on clinical expert criteria, utilizing faster region-based convolutional neural networks (Faster R-CNN) for the classification of tongue images. Faster R-CNN achieved strong performance, with average accuracy, recall, precision, and F1-score reaching 90.67%, 91.25%, 99.28%, and 95.00%, respectively. Applied to a medical screening population, the Faster R-CNN model detected various tongue features, with cracked tongue (41.49%) and teeth-marked tongue (37.16%) being the most prevalent [144]. Based on convolutional neural network (CNN) technology, Wang et al. developed the Greasy Tongue Coating Recognition Networks (GreasyCoatNet) using a dataset of 1,486 tongue images as standard references. This innovative approach offers a new paradigm for symptom differentiation and personalized intervention strategies [145]. Besides, *palpation* is one of the primary methods used by TCM practitioners for disease diagnosis. AI technology could facilitate pulse classification and disease prediction by collecting pulse signals in clinical settings and from medical literature (Table 7). Luo et al. used ML to predict and classify pulse waves in hypertensive and healthy groups. The models achieved high accuracy for hypertension classification using questionnaire and pulse wave data, with performance enhanced after K-means noise removal: AdaBoost and Gradient Boosting reached 86.41% accuracy (AUC 0.86), Random Forest achieved 85.33% (AUC 0.85), and SVM notably improved from 79.57% to 83.15% accuracy (AUC stability: 0.79 to 0.83)[146]. Yan et al. utilized DCNN kernel techniques to extract features from pulse signals and established a classification model based on SVM. This approach enhanced the objectivity and accuracy of pulse diagnosis, achieving an average accuracy of 0.8330 using ensemble learning [147]. Additionally, the multi-label learning (MLL) technique could be applied to auscultation and interrogation, facilitating the building of standardized inquiry models and enhancing the level of personalized diagnosis [9, 148].

**Table 7:** AI-powered syndrome differentiation in TCM

Systems		Training data	Algorithms	Application	Refs.
	Knowledge discovery-based	EMRs	Collaborative neighbor algorithm	Differentiation of tinnitus symptoms	[142]
			Bayesian and XGBoost	Metabolic syndrome prediction	[149]
TCMSSD		Syndromes	DL, KG, BM25 algorithm	Precise diagnoses, personalized treatment plans	[26]
		Characteristics of individual physical examination data	Logistic regression, bayesian network, decision tree	Association between metabolic syndrome and TCM constitution	[150]
F-MT-KER		TCM characteristics	KG, BERT	TCM syndrome differentiation of complex cases	[143]
		Heart sound data collected in the clinic and from medical books	CNN	Heart sound recognition	[151]
Faster R-CNN	*Inspection*	Tongue images	DL	Tongue image classification	[144]
GreasyCoatNet			Deep transfer learning	Assist syndrome diagnosis, ethnopharmacological evaluation	[145]
		Four lip images	SVM-RFE	Quantitative examination on lip diagnosis of TCM	[152]
	*Palpation*	Pulse wave information	K-means	Pulse diagnosis in TCM	[146]
		Pulse signal	SVM, DCNN	Classification and recognition of pulse signal	[147]
DeepFHR		Fetal heart rate signals	CNN	A forecast for fetal acidemia	[153]
	*Auscultation*	Patients'speech samples of 5 vowels	MIML technique	Auscultation analysis	[148]
		Voice signals	Wavelet packet transform and sample entropy		[154]
	*Interrogation*	CHD inquiry diagnosis cases	MLL	CHD diagnosis	[9]

F-MT-KER: Fused medical text and KG entity representation; Faster R-CNN: Faster region-based convolutional neural networks; GreasyCoatNet: Greasy tongue coating recognition networks; EMRs: Electronic medical records; KG: Knowledge graph; BERT: Bidirectional encoder representations from transformers; SVM: support vector machine; DCNN: convolutional neural network; MIML: multi-instance multi-label; MLL: Multi-label learning technique; CHD: Coronary heart disease

### Herb combinations/TCM prescriptions recommendation

TCM formulations typically include “*Monarch medicine*”, “*Minister medicine*”, “*Adjuvant medicine*” and “*Envoy medicine*”. This multi-layered structure embodies the holistic approach and personalized treatment philosophy of TCM. However, this also makes the pharmacology of TCM more complex, because it is difficult to explain the complex relationships or synergies of the parts.

#### NPM-based herb combinations

While NPM has enabled the effective screening of herbal formulas for specific diseases, developing universal methods for recommending and generating TCM prescriptions continues to pose a significant challenge in TCM knowledge discovery [155, 156]. Fortunately, recent advances in AI have enabled the development of methods that integrate TCM data with DL/ML, KG, and network-based techniques (Table 8). These approaches are being applied to areas such as TCM-symptom interaction analysis, TCM-modern pharmacological mechanism exploration, and TCM-electronic health records (EHRs) interactions, among others. For example, the network-based method facilitates the construction of a PPI network for a given pair of TCM, enabling the quantification of interactions between the herb pairs and their target proteins. Through the analysis of synergistic compound interactions within the network topology, this approach enhanced the exploration of potential herbal combinations [155, 157, 158].

**Table 8:** Building an AI-powered system for TCM herbal compatibility analysis and prescription recommendation

Systems	Classic	Training data	Model	Refs.
Network-based forecasting	Chinese herbal compatibility	Herb-symptom-target	Network proximity	[157]
		Herbs-ingredients-targets	Multiple network-based distances	[158]
		Prescription-herbs-ingredients-targets	DMIM	[155]
Omics based approach		Metabolomics data		[165]
TCMM	Prescription recommendation	Symptoms	KG augmentation	[42]
TCMPR		Clinical symptom terms	Subnetwork-based symptom term mapping method	[166]
		Symptoms	MGCN	[8]
SMRGAT		Symptoms'semantic information and external knowledge of herbs	Multi-graph residual attention network and semantic knowledge fusion	[167]
HPE-GCN		Classic tonic formulae	GCN	[168]
TCMFP		Traditional experiences and modern pharmacological mechanisms	Semi-supervised learning genetic algorithms	[169]
OSPF		*Hot* and *cold* formulae	ANN	[10]
		Symptom/herb feature representations	GCN	[170]
FordNet		20,000 electronic health records	DNN	[162]
SoFDA/ETCM 2.0		Chinese patent drugs, Chinese medicinal materials	Jaccard and cosine similarity	[4, 161]
SymMap		Herbal constituents, target genes	Statistical testing	[37]
		Facial and tongue images	Two-stream vision transformer based multi-label recognition	[164]
		Tongue images	Single/double convolution channels	[171]
DGSCAM		Symptoms, clinical dataset	Knowledge source guidance network	[163]
		Clinical symptoms, Sasang constitution types, TCM prescriptions	LIME technique	[172]
DMRNet		EMRs, Patient characteristics	KG	[173]

KG: Knowledge graph; MGCN: Multigraph convolutional network; GCN: Graph convolutional networks; DNN: Deep neural network; DMIM: Distance-based mutual information model; ANN: Artificial neural network; LIME: Local interpretable model-agnostic explanation; DGSCAM: dual-branch guidance strategy combined with candidate attention model

#### Herbal formula recommendation based on real-world experience

TCM is a medical system that emphasized individualized treatment, formulating personalized therapeutic plans based on the specific symptoms and individual differences of patients. Chinese herbal formulas can be flexibly adjusted according to the particular circumstances of the patient, aiming to achieve optimal clinical efficacy. Therefore, generating prescriptions based on patient characteristics and clinical outcomes played a critical role in improving the health status of patients [159, 160]. The integration of multi-dimensional data of TCM databases with ML and DL techniques has significantly advanced TCM research, development, and assisted diagnosis (Table 8). Platforms such as SoFDA and ETCM 2.0 utilized jaccard and cosine similarity calculations to support disease diagnosis and recommend prescriptions/herbs/ingredients with similar clinical efficacy [4, 161]. Similarly, SymMap applied statistical testing to rank and filter promising components based on their therapeutic potential [37]. Meanwhile, DL and ML models integrated TCM knowledge bases, symptom information, and clinical data, employing embedding techniques to extract and represent knowledge base information, leveraging NLP to learn and achieve the quantitative expression of diverse TCM elements. Ultimately, by selecting the model with the highest accuracy and best performance, they enable precise prescription recommendations. For example, Zhou et al. introduced a DL-based prescription recommendation system FordNet, which extracts phenotypic information from over 20,000 EHRs. By utilizing embedding techniques, FordNet captured the characteristics of TCMs from a heterogeneous network that includes TCMs, prescriptions, and molecular targets. The system integrated this phenotypic and molecular information to provide comprehensive insights. Clinical evaluations have demonstrated that FordNet effectively learns from the valuable experience of TCM practitioners and offers robust recommendations for new prescriptions [162].

Moreover, integrating symptom text data with clinical datasets could enhance the professionalism of prescription generation. For example, TCMM database integrated KG augmentation and multi-hop reasoning to generate TCM prescriptions and symptom-related target prediction [42]. In addition, Hou et al. utilized dual-branch guidance strategy combined with candidate attention model (DGSCAM) model to extract critical information from'symptom-prescription'data pairs and conducted extensive experiments on both public and clinical datasets. The results demonstrated that the prescriptions generated by DGSCAM were more professional compared to those from baseline models [163]. Chinese medicine doctors prescribed Chinese medicine through the dialectic of symptoms, including the diagnosis of facial and tongue images. Zhao et al. proposed a multi-herb recommendation framework based on visual transformers and multi-label classification. By conducting experiments with real facial and tongue images, this framework generated prescription data that closely resembles authentic samples [164].

## TCM generative pre-trained transformer

**Fig. 10:**
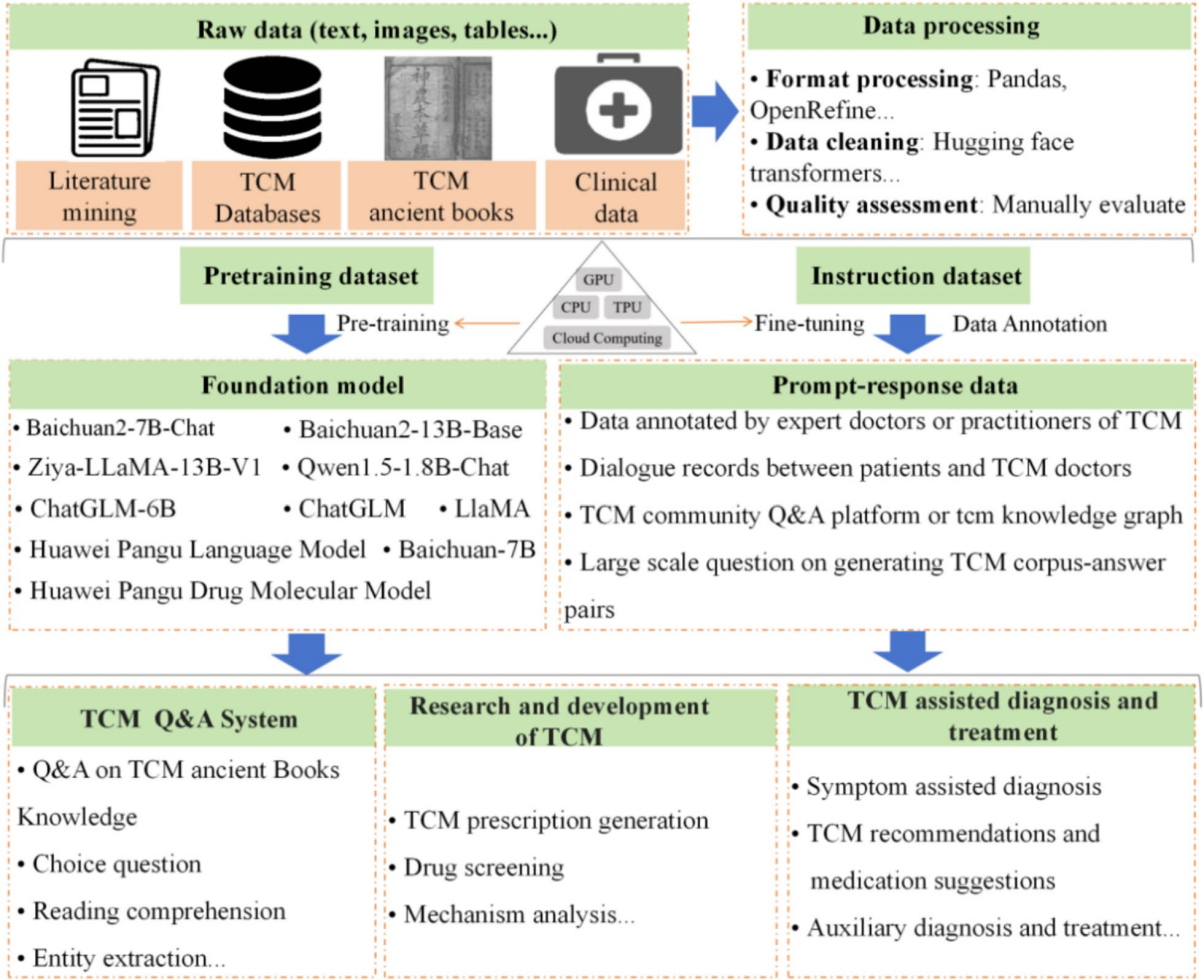
Architecture diagram of TCM LLMs

TCM involved a vast and intricate body of knowledge, including ancient texts, clinical data, experimental evidence, and target signaling pathway, etc. The integration of TCM knowledge with modern science through LLMs represented a groundbreaking development in the research and treatment of TCM, such as TCMChat, Lingdan and MedChatZH (Table 9, Appendix 4). Specifically, the rise of AI technologies, especially large pre-trained (PT) models (e.g., GPT1/2[174], BERT [175], Roberta [176], T5 [177] and DeBERTa [178], are trained on a large generic corpus, acquiring a wide range of language knowledge, and can then be fine-tuned (FT) to suit specific NLP scenarios, such as question answering systems, machine translation, and human–computer interaction (Fig. 10). Meanwhile, by leveraging the PT and instruction FT paradigm, along with retrieval-augmented generation (RAG) techniques, these models are capable of extracting and generating TCM knowledge with high accuracy. This approach enabled LLMs to effectively answer complex questions about TCM and significantly reduced the amount of data and compute resources, such as GPUs and CPUs, required to retrain the model for each new task. For example, TCMChat used the customized set of 6 TCM scenarios of large-scale TCM text knowledge as the training set, takes Baichuan 2-7b-chat as the base model, and carries out PT and SFT processing on the model, providing a high-quality knowledge base for TCM modernization research [12]. Based on three key data sets of ancient Chinese medicine books, textbooks and clinical data, the Lingdan-TCM-CHAT and Lingdan-PR model were fine-tuned to improve the performance and accuracy of Lingdan-PT LLM in answering questions and recommending TCM [13]. Besides, Huang-Di model warehouse and ZhongJing TCM model realized the medical dialogue between TCM and ancient books by pre-training ZiA-LLAMA 13B-V1 and Qwen1.5–1.8B-Cha respectively (Appendix 4).

**Table 9:** Introduction of existing LLMs for TCM

TCM large language models	Full name	Foundation model	Instruction data	Training paradigm	Model evaluation	Scenarios	Refs.
TCMChat	LLMs	Baichuan2-7B-Chat	4 national standards, 7 medical textbooks, 18 medical cases	PT, SFT	Accuracy, BLEU, Meteor, ROUGE-1, ROUGE-2, ROUGE-L, BertScore	TCM knowledgebase, choice question, reading comprehension, entity extraction, medical case diagnosis, and herb or formula recommendation	[12]
Lingdan	Lingdan-TCPM-Chat and Lingdan-PR	Baichuan2-13B-Base	TCM ancient books, textbooks, and clinical data	PT, SFT	F1-score	Q&A on TCM clinical knowledge and recommendations for herbal prescriptions	[13]
MedChatZH	Large Language Model Meta AI	Baichuan-7B	1000 TCM books	PT, FT	BLEU, GLEU, ROUGE-1, ROUGE-2, and ROUGE-L	Q&A and dialogue systems	[14]
OpenTCM		GraphRAG-empowered	68 gynecological books	PT, FT	Precision, Recall, F1-Score, Accuracy	TCM information search and intelligent Q&A	[181]
CPMI-ChatGLM		ChatGLM-6B	3906 annotated Chinese patent drug records	FT	BLEU, ROUGE, and BART Score	TCM recommendations and medication suggestions	[179]
ACUBERT		BERT model	54,593 different entities from 82 acupuncture medical books	SVM, RF	Precision, recall, F1 scores	Meridian entity recognition and classification	[180]
ZhongJing TCM model		LLaMA	Proprietary medical dataset	PT, SFT, RLHF		TCM Q&A	[182]

DPO: Direct preference optimization; LLMs: Ground-breaking large language models; RLHF: Reinforcement learning from human feedback; PT: pre-trained; FT: fine-tuned; SFT: Supervised Fine-Tuning; RLHF: Reinforcement learning from human feedback; Q&A: Question-Answering; BLEU: Bilingual evaluation understudy; Meteor: Metric for evaluation of translation with explicit ordering; ROUGE-1/2/L: Recall-oriented understudy for gisting evaluation; BertScore: BERT-based evaluation score; SVM: support vector machine; RF: random forest

In addition, LLMs performed well in PT and FT based on real-world data sets for the application of TCM in assisted diagnosis and treatment. For instance, based on the transformer decoder with the LLaMA architecture, MedChatZH outperformed several Chinese dialogue baselines on a real-world medical dialogue dataset using ROUGE-1, ROUGE-2, and ROUGE-L to evaluate the quality of model responses based on word matches in the longest common subsequence. This achievement is credited to its PT on Chinese medical literature, followed by FT with a meticulously selected medical instruction dataset [14]. Compared with Chinese-LLaMA-7B, Chinese-Alpaca-7B, Qwen-7B, and Baichuan-7B, CPMI-ChatGLM achieves the best performance in supporting diagnostic and treatment tasks, with ROUGE-1, ROUGE-2, ROUGE-L, BLEU-4, and BARTScore values of 0.8188, 0.7738, 0.8107, 0.7641, and −2.4786, respectively [179]. And the LLMs Lingdan and ACUBERT demonstrated strong performance in TCM recommendations and medication suggestions [13, 180].

These capabilities of LLMs not only deepened the practical utility of TCM but also enhanced its accessibility and integration into modern medical practices. Meanwhile, the increasing global interest in TCM also highlighted the need for modern, evidence-based tools to support its internationalization and scientific validation, making large LLMs a crucial step forward.

## Challenges

The deep integration of AI technology with the theories, knowledge, and clinical practices of TCM remains the primary challenge in the modernization of TCM. AI faces core challenges in addressing the subjectivity and holism of TCM, mainly includes scarcity of high-quality data, heterogeneity, interpretability, ethical issues, data privacy issues, and clinical specificity of data. In addition, the application of AI in clinical training, technology adoption, and integration with existing healthcare still needs to be further strengthened [2].

### Difficulty in standardizing multimodal data of TCM

Currently, the multimodal data of TCM faces the following challenges: 1) Difficulty in multimodal data fusion. The data in the field of TCM is highly complex and inherently inconsistent in terms of sources, structure, semantics, knowledge systems, and clinical applications. For example, structured clinical data aligned with TCM logic are rare, and classical texts are largely unstructured, severely constraining model training efficacy; 2) Quantifying subjective experience. TCM diagnosis relies on practitioners'personal intuition, yet such subjective insights resist standardization for AI learning; 3) Privacy protection presents critical hurdles in handling TCM's multi-modal datasets, as hospitals strictly limit access to these identifiable health records [183].

### The complexity of clinical practice

(1) Complexity of holistic correlation. TCM emphasizes dynamic equilibrium among organs, *qi*-blood, and *yin*-*yang*, requiring comprehensive syndrome differentiation based on systemic conditions. AI struggles to capture multidimensional nonlinear relationships, often simplifying analyses into local segments, and can only reflect the subjectivity and holism of TCM to a limited extent [184]; (2) Limitations in personalized treatment. The principle “different treatments for the same disease, same treatment for different diseases” demands dynamic adjustments based on individual constitutions. Current AI lacks adaptability, tending toward standardized rather than personalized outcomes [156]; (3) Current AI models fail to dynamically adapt to patients'individual constitutions, rely solely on clinical data from select hospitals. And AI technologies cannot reflect the synergistic interactions among genes, body constitution, and environmental factors. Moreover, the interpretability of AI predictions remained a concern, especially when it comes to regulatory approval and clinical acceptance [2].

### Moral and ethical issues of AI

In the practice of TCM diagnosis and TCM development, AI models leverage vast clinical datasets and electronic health records to provide functions such as herbal formula recommendations and diagnostic support. Since the high-quality multi-modal data originates from patients, this necessitates robust privacy protection measures and ethically-grounded guidelines[185].

## Future directions

The integration of AI into TCM multi-scale data has opened new horizons for both research and clinical applications. In this study, (1) The application of multimodal data in TCM within the field of AI enhanced the precision and scientific rigor of TCM research by integrating information across multiple levels, from micro to macro. AI technologies not only deciphered the inherent patterns of TCM's complex systems but also drove data-driven innovative research, intelligent decision support, and interdisciplinary collaboration; (2) Research technologies driven by AI, such as ML, DL, multi-omics, and NPM, are being applied to various aspects of TCM, including target discovery for NPs, VS of NPs, quality control of TCM, and personalized prescription recommendations; (3) AI technology have enhanced the objectivity and standardization of TCM diagnosis. By analyzing real-world data, it optimized chronic disease management and promoted personalized TCM treatment, thereby more accurately and effectively improving treatment outcomes; (4) The application of LLMs demonstrated remarkable efficiency in data processing, swiftly uncovering critical insights from vast repositories of TCM multimodal data. This capability supported the optimization of herbal prescriptions, advances in pharmacological studies, and the discovery of potential therapeutic effects, thereby providing robust support for the modernization of TCM. Furthermore, these models excelled at aiding diagnostics by speeding up the process, improving accuracy, and recommending personalized treatment plans, which enhanced therapeutic outcomes.

In the future, the strengthening of PT and integration of TCM knowledge is essential, achieved through the construction of specialized TCM PT corpora to enhance AI models'comprehension of professional terminology and domain-specific knowledge. Further leveraging generative AI to parse unstructured data from TCM classical texts, clinical case records, and medical histories, transforming them into standardized digital information to overcome bottlenecks in multi-modal TCM data integration [186–188]. Then, establishing a reasoning and explanation mechanism based on knowledge graphs, we will create a system highly compatible with TCM knowledge, advancing explainable AI technology to achieve transparency in the decision-making process. By introducing retrieval-augmented generation (RAG) technology, we will integrate LLMs with multimodal data retrieval, forming a closed-loop processing mechanism of"retrieval-reasoning-generation". This approach reduces the model’s probability of hallucination while enhancing its performance and trustworthiness [189, 190]. Concurrently, ethical guidelines and management systems for AI technology applications should be formulated to ensure the orderly integration of technological innovation while safeguarding patient privacy [191]. Furthermore, developing the integration of digital twin technology and causal reasoning with TCM practice involves creating personalized virtual models for each patient. Through the continuous integration of real-time monitoring data, these models are dynamically updated. Causal reasoning can then analyze the causal relationships between specific interventions and health outcomes within the individual, predicting the potential effects and risks of different treatment plans for that specific patient, thereby achieving authentic precision Chinese medicine [192].

## Conclusions

The application of AI in TCM research and clinical practice offered transformative opportunities. In the future, by harnessing the power of AI, we could unlock the full potential of TCM, paving the way for innovative therapies and personalized healthcare solutions that benefit patients worldwide. The continued collaboration between AI experts and TCM practitioners will be crucial in realizing these advancements and ensuring the harmonious integration of modern technology with ancient TCM wisdom.

## Supplementary Information

Supplementary Material 1

Supplementary Material 2

Supplementary Material 3

Supplementary Material 4

Supplementary Material 5

## Data Availability

The data and materials used in this study are entirely based on publicly available datasets and information obtained through literature reviews. All datasets utilized can be accessed from their respective sources without restrictions. Specific sources and references have been detailed within the manuscript to facilitate access for interested researchers. Therefore, all data and materials supporting the results of this study are freely available to any scientist wishing to use them for non-commercial purposes, without breaching participant confidentiality.

## Abbreviations

ABPP: Activity-based protein profiling
ABPs: Activity-based probes
AD: Alzheimer's disease
ADME: Absorption, distribution, metabolism, excretion
AI: Artificial intelligence
Akt: Serine/threonine kinase
ANN: Artificial neural network
BBB: Blood-brain barrier
BERT: Bidirectional encoder representations from transformer
BertScore: BERT-based evaluation score
BLEU: Bilingual evaluation understudy
CCCP: Compound-centric chemical proteomics
CETSA: Cellular thermal shift assay
CHD: Coronary heart disease
CMap: Connectivity map
CNN: Convolutional neural network
CRISPR: Clustered regularly interspaced short palindromic repeats
CSE1L: Exportin-2
DARTS: Drug affinity responsive target stability
DCNN: Convolutional neural network
DGSCAM: Dual-branch guidance strategy combined with candidate attention model
DL: Deep learning
DL: Drug-likeness
DMIM: Distance-based mutual information model
DNN: Deep neural network
DPO: Direct preference optimization
ECFP: Extended-connectivity fingerprint
EMRs: Electronic medical records
Faster R-CNN: Faster region-based convolutional neural networks
FMh2v: Cross-feature-based unsupervised pretraining model
F-MT-KER: Fused medical text and KG entity representation
GC: Gas chromatography
GCN: Graph convolutional networks
GreasyCoatNet: Greasy tongue coating recognition networks
HL: Half-life
HPLC: High-performance liquid chromatography
HPO: Human phenotype ontology
KG: Knowledge graph
LBVS: Ligand-based virtual screening
LIME: Local interpretable model-agnostic explanation
LLMs: Large language models
MD: Molecular dynamics simulations
Meteor: Metric for evaluation of translation with explicit ordering
MGCN: Multigraph convolutional network
MHC-I: Major histocompatibility complex 1
MIML: Multi-instance multi-labe
ML: Machine learning
MLL: Multi-label learning
MLP: Multilayer perceptron
MS: Mass spectrometry
MW: Molecular weight
NF-κB: Nuclear factor kappa-B
NLP: Natural language processing
NPM: Network pharmacology
NPs: Natural products
Nrf2: Uclear factor erythroid 2-related factor 2
OB: Oral bioavailability
OMIM: Online mendelian inheritance in man
OSPF: Ontology-based side-effect prediction framework
PI3K: Phosphatidylinositol 3-kinase
PRISMA: Preferred reporting items for systematic reviews and meta-analyses
PT: Pre-training
Q&A: Question-answering
Q-marker: Quality markers
QSAR: Quantitative structure-activity relationship
RAG: Retrieval-augmented generation
RF: Random forest
RG: Gyration
RLHF: Reinforcement learning from human feedback
RMSD: Root mean square deviation
ROUGE-1/2/L: Recall-oriented understudy for gisting evaluation
SBVS: Structure-based virtual screening
SDS: Sarsasapogenin derivatives
SFT: Supervised fine-tuning
SPROX: Stability of proteins from rates of oxidation
STAT3: Signal transducer and activator of transcription 3
SVM: Support vector machine
SysDT: Systems dynamics and therapeutics
TCM: Traditional Chinese medicine
TF-IWF: Term frequency-inverse word frequency
TPP: Thermal proteome profiling
