# Supplementary material for: Advancing the modernization of traditional Chinese medicine through artificial intelligence and multimodal data integration
Source: Chin Med. 2026 Jan 26;21:54. doi: 10.1186/s13020-025-01194-y (PMC12833950; doi:10.1186/s13020-025-01194-y)
Supplement: Supplementary file 1 — Supplementary Material 1 [file 13020_2025_1194_MOESM1_ESM.docx]

**Table 1.** Multiscale data resources for TCM

| **Database** | **Prescriptions** | **Herbs** | **Ingredients** | **Targets** | **Diseases** | **Syndromes** | **Clinical records** | **Total** | **Effect** | **Ref.** |
| --- | --- | --- | --- | --- | --- | --- | --- | --- | --- | --- |
| LTM-TCM | 48,126 | 9122 | 34,967 | 13,109 | / | 1928 | 41025 | 148277 | Linking of TCM with modern medicine at molecular and phenotypic levels; drug discovery | [1] |
| ETCM 2.0 | 48,442 | 2079 | 38,298 | 1040 | 8045 | 319 | / | 98223 | Mechanistic research; drug discovery; target identification; quality marker identification | [2] |
| TCMSSD | 133518 | 8259 | 43413 | 17602 | 8073 | 624 | / | 211489 | Syndrome standardization; syndrome prediction | [3] |
| TCMM | 48043 | 8932 | 69816 | 76449 | 22365 | 1900 | / | 227505 | Extensive TCM modernization knowledge; TCM modernization and therapeutic innovations | [4] |
| TM-MC 2.0 | 5075 | 635 | 34107 | 13992 | 27997 | / | / | 81806 | Compound screening; drug discovery | [5] |
| TCM-Suite | 6,692 | 7,322 | 704,321 | 19,319 | 15,437 | / | / | 753091 | TCM component identification; network pharmacology analysis | [6] |
| TCMID 2.0 | 15 | 778 | 18203 | 82 | 842 | / | / | 19920 | TCM's modernization; exploring of underlying biological processes | [7] |
| SymMap | / | 499 | 19595 | 4302 | 5235 | 1717 | / | 31348 | Symptom mapping; phenotypic drug discovery | [8] |
| CPMCP | / | 1557 | 26341 | 20965 | 14086 | 2285 | / | 65234 | Standardized TCM symptom; associations between TCM symptoms and MM symptoms | [9] |
| TCMIO | 1493 | 618 | 126973 | 154 | / | / | / | 129238 | Molecular mechanisms of TCM in modulating the cancer immune microenvironment | [10] |
| YaTCM | 1813 | 6,220 | 47,696 | 18,697 | / | / | / | 74426 | Identify the potential ingredients;investigate the mechanism of action for TCM; predict potential targets for TCM molecules; explore functionally similar herb pairs | [11] |
| TCMSTD | 22 | 252 | 4361 | 2425 | / | / | / | 7060 | A systematic analysis of the traditional Chinese medicine system toxicology | [12] |
| TCM-ADIP | 2100 | 744 | 23524 | 17176 | / | / | / | 43544 | Linking TCM to functional brain zones of Alzheimer's disease | [13] |
| TCM-Mesh | / | 6235 | 383840 | 14298 | 6204 | / | / | 410577 | Drug combinations; understand the underlying mechanisms for TCM | [14] |
| TCMBank | / | 9192 | 61966 | 15179 | 32529 | / | / | 118866 | DL-based Chinese-Western medicine exclusion prediction | [15] |
| HERB | / | 7263 | 49258 | 12933 | 28212 | / | / | 97666 | A high-throughput experiment- and reference-guided database; modern drug discovery | [16] |
| TCMSP | / | 499 | 29384 | 3311 | 837 | / | / | 34031 | Identify drug-target networks/drug-disease networks; uncovering TCM theory; developing herb-oriented drugs | [17] |
| BATMAN-TCM 2.0 | 54832 | 8404 | 39171 | / | / | / | / | 102407 | Expanded ingredient-target interaction; TCM molecular mechanisms; developing new drugs | [18] |
| TCM2COVID | 280 | 300 | 80 | / | / | / | / | 660 | Anti-COVID-19 traditional Chinese medicine with effects and mechanisms | [19] |
| DCABM-TCM | 192 | 194 | 1816 | / | / | / | / | 2202 | TCM molecular mechanism elucidation; screening of candidate blood constituents | [20] |
| TCMSID | / | 499 | 20015 | 3270 | / | / | / | 23784 | Implementing multi-tool target prediction; drug discovery | [21] |
| HIT 2.0 | / | 1250 | 1237 | 2208 | / | / | / | 4695 | Herbal ingredients' targets based on literature mining | [22] |
| [TCM@Taiwan](mailto:TCM@Taiwan) | / | 453 | 20000 | / | / | / | / | 20453 | Drug screening | [23] |

Reference:

1. Li X, Ren J, Zhang W, Zhang Z, Yu J, Wu J, Sun H, Zhou S, Yan K, Yan X *et al*: **LTM-TCM: A comprehensive database for the linking of Traditional Chinese Medicine with modern medicine at molecular and phenotypic levels**. *Pharmacol Res* 2022, **178**:106185.

2. Zhang Y, Li X, Shi Y, Chen T, Xu Z, Wang P, Yu M, Chen W, Li B, Jing Z *et al*: **ETCM v2.0: An update with comprehensive resource and rich annotations for traditional Chinese medicine**. *Acta Pharm Sin B* 2023, **13**(6):2559-2571.

3. Huang L, Wang Q, Duan Q, Shi W, Li D, Chen W, Wang X, Wang H, Chen M, Kuang H *et al*: **TCMSSD: A comprehensive database focused on syndrome standardization**. *Phytomedicine* 2024, **128**:155486.

4. Ren Z, Ren Y, Li Z, Xu H: **TCMM: A unified database for traditional Chinese medicine modernization and therapeutic innovations**. *Comput Struct Biotechnol J* 2024, **23**:1619-1630.

5. Kim SK, Lee MK, Jang H, Lee JJ, Lee S, Jang Y, Jang H, Kim A: **TM-MC 2.0: an enhanced chemical database of medicinal materials in Northeast Asian traditional medicine**. *BMC Complement Med Ther* 2024, **24**(1):40.

6. Yang P, Lang J, Li H, Lu J, Lin H, Tian G, Bai H, Yang J, Ning K: **TCM-Suite: A comprehensive and holistic platform for Traditional Chinese Medicine component identification and network pharmacology analysis**. *Imeta* 2022, **1**(4):e47.

7. Huang L, Xie D, Yu Y, Liu H, Shi Y, Shi T, Wen C: **TCMID 2.0: a comprehensive resource for TCM**. *Nucleic Acids Res* 2018, **46**(D1):D1117-d1120.

8. Wu Y, Zhang F, Yang K, Fang S, Bu D, Li H, Sun L, Hu H, Gao K, Wang W *et al*: **SymMap: an integrative database of traditional Chinese medicine enhanced by symptom mapping**. *Nucleic Acids Res* 2019, **47**(D1):D1110-d1117.

9. Sun C, Huang J, Tang R, Li M, Yuan H, Wang Y, Wei JM, Liu J: **CPMCP: a database of Chinese patent medicine and compound prescription**. *Database (Oxford)* 2022, **2022**.

10. Liu Z, Cai C, Du J, Liu B, Cui L, Fan X, Wu Q, Fang J, Xie L: **TCMIO: A Comprehensive Database of Traditional Chinese Medicine on Immuno-Oncology**. *Front Pharmacol* 2020, **11**:439.

11. Li B, Ma C, Zhao X, Hu Z, Du T, Xu X, Wang Z, Lin J: **YaTCM: Yet another Traditional Chinese Medicine Database for Drug Discovery**. *Comput Struct Biotechnol J* 2018, **16**:600-610.

12. Song L, Qian W, Yin H, Sun Y, Sun X, Li G, He J, Zheng Y, Zhang Y, Wang J *et al*: **TCMSTD 1.0: a systematic analysis of the traditional Chinese medicine system toxicology database**. *Sci China Life Sci* 2023, **66**(9):2189-2192.

13. Hu L, Tang Q, Meng F, Xu Y, Chen W, Xu S: **TCM-ADIP: A Multidimensional Database Linking Traditional Chinese Medicine to Functional Brain Zones of Alzheimer's Disease**. *J Mol Biol* 2024:168874.

14. Zhang RZ, Yu SJ, Bai H, Ning K: **TCM-Mesh: The database and analytical system for network pharmacology analysis for TCM preparations**. *Sci Rep* 2017, **7**(1):2821.

15. Lv Q, Chen G, He H, Yang Z, Zhao L, Zhang K, Chen CY: **TCMBank-the largest TCM database provides deep learning-based Chinese-Western medicine exclusion prediction**. *Signal Transduct Target Ther* 2023, **8**(1):127.

16. Fang S, Dong L, Liu L, Guo J, Zhao L, Zhang J, Bu D, Liu X, Huo P, Cao W *et al*: **HERB: a high-throughput experiment- and reference-guided database of traditional Chinese medicine**. *Nucleic Acids Res* 2021, **49**(D1):D1197-d1206.

17. Ru J, Li P, Wang J, Zhou W, Li B, Huang C, Li P, Guo Z, Tao W, Yang Y *et al*: **TCMSP: a database of systems pharmacology for drug discovery from herbal medicines**. *J Cheminform* 2014, **6**:13.

18. Kong X, Liu C, Zhang Z, Cheng M, Mei Z, Li X, Liu P, Diao L, Ma Y, Jiang P *et al*: **BATMAN-TCM 2.0: an enhanced integrative database for known and predicted interactions between traditional Chinese medicine ingredients and target proteins**. *Nucleic Acids Res* 2024, **52**(D1):D1110-d1120.

19. Ren L, Xu Y, Ning L, Pan X, Li Y, Zhao Q, Pang B, Huang J, Deng K, Zhang Y: **TCM2COVID: A resource of anti-COVID-19 traditional Chinese medicine with effects and mechanisms**. *Imeta* 2022, **1**(4):e42.

20. Liu X, Liu J, Fu B, Chen R, Jiang J, Chen H, Li R, Xing L, Yuan L, Chen X *et al*: **DCABM-TCM: A Database of Constituents Absorbed into the Blood and Metabolites of Traditional Chinese Medicine**. *J Chem Inf Model* 2023, **63**(15):4948-4959.

21. Zhang LX, Dong J, Wei H, Shi SH, Lu AP, Deng GM, Cao DS: **TCMSID: a simplified integrated database for drug discovery from traditional chinese medicine**. *J Cheminform* 2022, **14**(1):89.

22. Yan D, Zheng G, Wang C, Chen Z, Mao T, Gao J, Yan Y, Chen X, Ji X, Yu J *et al*: **HIT 2.0: an enhanced platform for Herbal Ingredients' Targets**. *Nucleic Acids Res* 2022, **50**(D1):D1238-d1243.

23. Chen CY: **TCM Database@Taiwan: the world's largest traditional Chinese medicine database for drug screening in silico**. *PLoS One* 2011, **6**(1):e15939.
