# Supplementary material for: Advancing the modernization of traditional Chinese medicine through artificial intelligence and multimodal data integration
Source: Chin Med. 2026 Jan 26;21:54. doi: 10.1186/s13020-025-01194-y (PMC12833950; doi:10.1186/s13020-025-01194-y)
Supplement: Supplementary file 2 — Supplementary Material 2 [file 13020_2025_1194_MOESM2_ESM.docx]

**Table 3.** AI-driven platform and database for natural product target prediction

| **Systems** | **Methods** | **Advantage** | **Limitation** | **Webserver** | **Ref.** |
| --- | --- | --- | --- | --- | --- |
| TCMSP | SysDT model | Multi algorithm fusion prediction (RF, SVM), Cross scale data integration (chemical, genomic, and pharmacological information) [1] | Relying on the coverage of existing knowledge base | http://sm.nwsuaf.edu.cn/lsp/tcmsp.php | [2] |
| ETCM 2.0 | 2D ligand similarity | Efficient and fast, suitable for orphan targets, high sensitivity | Similar structure ≠ similar activity, neglecting the complexity of structure-activity relationships | http://www.tcmip.cn/ETCM2/front/ | [3] |
| YaTCM | Multi-voting chemical similarity | Revealing the binding mechanism, Discovering a brand new target, high specificity | Insufficient flexibility in handling, deviation of scoring function | http://cadd.pharmacy.nankai.edu.cn/yatcm/home | [4] |
| SuperPred 3.0 | Structural similarity of ECFP molecular fingerprint |  |  | <https://prediction.charite.de/subpages/target_prediction.php> | [5] |
| SwissTargetPrediction | The similarity of 2D and 3D structures |  |  | <http://swisstargetprediction.ch/> | [6] |
| TargetHunter | The similarity of 2D of known compounds |  |  | <https://www.cbligand.org/TargetHunter/login.php> | [7] |
| GalaxySagittarius-AF | Structur based |  |  | https://galaxy.seoklab.org/sagittarius_af | [8] |
| 3DSTarPred | 3D shape similarity | Multi conformational overlap similarity | Ignore chemical details, shape similarity ≠ combination similarity | https://3dstarpred.pumc.wecomput.com | [9] |
| LigandScout | Reversed pharmacophore matching | Breaking through the dilemma of "orphan ligands", high mechanism interpretability, multi target synergistic effect, high resource efficiency | Dependent on the quality of the target pharmacophore library, natural product specificity error | <http://www.inteligand.com/pharmdb/> | [10] |
| PharmMapper |  |  |  | http://lilab.ecust.edu.cn/pharmmapper/ | [11] |
| TCMIO/bSDTNBI | Balanced substructure-drug-target network-based inference | Prediction of synergistic targets of multiple components in TCM, high noise resistance and data compensation capability, balance the importance weight of target points | Subjectivity of substructure definition, insufficient dynamic control modeling | <http://lmmd.ecust.edu.cn/netinfer> | [12, 13] |
| CMap | Drug-induced gene expression profile | Directly associated phenotype, breaking through the limitations of the knowledge base, revealing dose dependence | Masking of cellular heterogeneity, complexity of data interpretation | <http://portals.broadinstitute.org/cmap/> | [14] |
| Lincscloud | Cell expression profile |  |  | <http://linscloud.org/> | [15] |
| DeepPurpose | DL | Breaking through the single representation of structure, high precision prediction, data utilization efficiency | Data dependency and bias amplification, interpretable black hole | https://github.com/kexinhuang12345/DeepPurpose | [16] |
| DeepDTA |  |  |  | https://github.com/hkmztrk/DeepDTA | [17] |
| DeepChem |  |  |  | https://pubmed.ncbi.nlm.nih.gov/?term=DeepChem | [18] |
| Interformer | Graph-transformer architecture | Dual modal feature fusion capability, dynamic weight adaptation, zero sample extrapolation potential | Computational complexity, interpretability gap | https://github.com/tencent-ailab/Interformer | [19] |
| ReduMixDTI | Feature redundancy reduction and interpretable attention mechanism | Reduction of feature redundancy, mechanism transparency, leap in computational efficiency | Information loss risk, attention misleading trap | https://github.com/mql430/ReduMixDTI. | [20] |
| CRISPR | Clustered regularly interspaced short palindromic repeats | The gold standard for causality verification, whole genome unbiased screening, compatibility of in *vitro* and in *vivo* models | Channel compensation problem, high technical threshold and cost | / | [21] |

**Label:** SysDT: Systems dynamics and therapeutics; RF: Random forest; SVM: Support vector machine; CRISPR: Clustered regularly interspaced short palindromic repeats

**Reference:**

1. Yu H, Chen J, Xu X, Li Y, Zhao H, Fang Y, Li X, Zhou W, Wang W, Wang Y: **A systematic prediction of multiple drug-target interactions from chemical, genomic, and pharmacological data**. *PLoS One* 2012, **7**(5):e37608.

2. Ru J, Li P, Wang J, Zhou W, Li B, Huang C, Li P, Guo Z, Tao W, Yang Y *et al*: **TCMSP: a database of systems pharmacology for drug discovery from herbal medicines**. *J Cheminform* 2014, **6**:13.

3. Zhang Y, Li X, Shi Y, Chen T, Xu Z, Wang P, Yu M, Chen W, Li B, Jing Z *et al*: **ETCM v2.0: An update with comprehensive resource and rich annotations for traditional Chinese medicine**. *Acta Pharm Sin B* 2023, **13**(6):2559-2571.

4. Li B, Ma C, Zhao X, Hu Z, Du T, Xu X, Wang Z, Lin J: **YaTCM: Yet another Traditional Chinese Medicine Database for Drug Discovery**. *Comput Struct Biotechnol J* 2018, **16**:600-610.

5. Gallo K, Goede A, Preissner R, Gohlke BO: **SuperPred 3.0: drug classification and target prediction-a machine learning approach**. *Nucleic Acids Res* 2022, **50**(W1):W726-w731.

6. Daina A, Michielin O, Zoete V: **SwissTargetPrediction: updated data and new features for efficient prediction of protein targets of small molecules**. *Nucleic Acids Res* 2019, **47**(W1):W357-w364.

7. Wang L, Ma C, Wipf P, Liu H, Su W, Xie XQ: **TargetHunter: an in silico target identification tool for predicting therapeutic potential of small organic molecules based on chemogenomic database**. *Aaps j* 2013, **15**(2):395-406.

8. Kwon S, Jung N, Yang J, Seok C: **GalaxySagittarius-AF: Predicting Targets for Drug-Like Compounds in the Extended Human 3D Proteome**. *J Mol Biol* 2024, **436**(17):168617.

9. Yan C, Liu Z, Bai Y, Wang Z, Fang J, Liu A: **3DSTarPred: A Web Server for Target Prediction of Bioactive Small Molecules Based on 3D Shape Similarity**. *J Chem Inf Model* 2024, **64**(21):8105-8112.

10. Wolber G, Langer T: **LigandScout: 3-D pharmacophores derived from protein-bound ligands and their use as virtual screening filters**. *J Chem Inf Model* 2005, **45**(1):160-169.

11. Wang X, Shen Y, Wang S, Li S, Zhang W, Liu X, Lai L, Pei J, Li H: **PharmMapper 2017 update: a web server for potential drug target identification with a comprehensive target pharmacophore database**. *Nucleic Acids Res* 2017, **45**(W1):W356-w360.

12. Liu Z, Cai C, Du J, Liu B, Cui L, Fan X, Wu Q, Fang J, Xie L: **TCMIO: A Comprehensive Database of Traditional Chinese Medicine on Immuno-Oncology**. *Front Pharmacol* 2020, **11**:439.

13. Zhou M, Sun J, Yu Z, Wu Z, Li W, Liu G, Ma L, Wang R, Tang Y: **Investigation of Anti-Alzheimer's Mechanisms of Sarsasapogenin Derivatives by Network-Based Combining Structure-Based Methods**. *J Chem Inf Model* 2023, **63**(9):2881-2894.

14. Yang K, Dinasarapu AR, Reis ES, Deangelis RA, Ricklin D, Subramaniam S, Lambris JD: **CMAP: Complement Map Database**. *Bioinformatics* 2013, **29**(14):1832-1833.

15. Duan Q, Flynn C, Niepel M, Hafner M, Muhlich JL, Fernandez NF, Rouillard AD, Tan CM, Chen EY, Golub TR *et al*: **LINCS Canvas Browser: interactive web app to query, browse and interrogate LINCS L1000 gene expression signatures**. *Nucleic Acids Res* 2014, **42**(Web Server issue):W449-460.

16. Huang K, Fu T, Glass LM, Zitnik M, Xiao C, Sun J: **DeepPurpose: a deep learning library for drug-target interaction prediction**. *Bioinformatics* 2021, **36**(22-23):5545-5547.

17. Öztürk H, Özgür A, Ozkirimli E: **DeepDTA: deep drug-target binding affinity prediction**. *Bioinformatics* 2018, **34**(17):i821-i829.

18. Zhang J, Zhao J, Lin H, Tan Y, Cheng JX: **High-Speed Chemical Imaging by Dense-Net Learning of Femtosecond Stimulated Raman Scattering**. *J Phys Chem Lett* 2020, **11**(20):8573-8578.

19. Lai H, Wang L, Qian R, Huang J, Zhou P, Ye G, Wu F, Wu F, Zeng X, Liu W: **Interformer: an interaction-aware model for protein-ligand docking and affinity prediction**. *Nat Commun* 2024, **15**(1):10223.

20. Liu M, Meng X, Mao Y, Li H, Liu J: **ReduMixDTI: Prediction of Drug-Target Interaction with Feature Redundancy Reduction and Interpretable Attention Mechanism**. *J Chem Inf Model* 2024, **64**(23):8952-8962.

21. Kasap C, Elemento O, Kapoor TM: **DrugTargetSeqR: a genomics- and CRISPR-Cas9-based method to analyze drug targets**. *Nat Chem Biol* 2014, **10**(8):626-628.
