# Supplementary material for: Advancing the modernization of traditional Chinese medicine through artificial intelligence and multimodal data integration
Source: Chin Med. 2026 Jan 26;21:54. doi: 10.1186/s13020-025-01194-y (PMC12833950; doi:10.1186/s13020-025-01194-y)
Supplement: Supplementary file 3 — Supplementary Material 3 [file 13020_2025_1194_MOESM3_ESM.docx]

**Table 5.** AI-based VS platform for NPs

| **Database** | **Classic** | **Methods** | **Advantage** | **Limitation** | **Ref.** |
| --- | --- | --- | --- | --- | --- |
| MTiOpenScreen | SBVS | AutoDock/AutoDock vina | High theoretical accuracy, efficiency and low cost, scaffold hopping, suitable for targets without ligand information, evaluate the stability of molecular dynamic simulation | Highly dependent on the quality of target structure, reliability of scoring function, difficulties in modeling solvation and entropy effects, incomplete coverage of chemical space, false positive and false negative risks | [1] |
| DockThor-VS |  | Multiple-solution genetic algorithm and the MMFF94S molecular force field |  |  | [2] |
| e-LEA3D |  | Genetic algorithm |  |  | [3] |
| Pharmit |  | Pharmacophores, molecular shape and energy minimization |  |  | [4] |
| PRODIGY-LIG |  | Making use of atomic instead of residue contacts |  |  | [5] |
| DOCKovalent |  | Covalent docking |  |  | [6] |
| AMMOS2 |  | Atomic-level energy minimization of a large number of experimental or modeled protein-ligand complexes |  |  | [7] |
| MedusaDock 2.0 |  | Incorporating structural constraints |  |  | [8] |
| AnchorQuery |  | Pharmacophore search |  |  | [9] |
| CRDS |  | Consensus reverse docking |  |  | [10] |
| RosettaVS |  | RosettaGenFF-VS | Simulate the motion of its flexible side chains and finite main chain | Incomplete coverage of chemical space | [11] |
| Roboticized AI |  | Microfluidic photocatalytic synthesis | Fully automated, high-throughput photocatalytic reaction, characterization, and screening system | Further improved to meet the needs of different types of photocatalytic reactions | [12] |
| ChemDes | LBVS | Computing molecular descriptors and fingerprints | No target structure required, extremely high computational efficiency, small-sample friendly, circumvents the protein flexibility challenge, and integrates multi-dimensional ligand information | Highly dependent on the quality of known activity data, limited scaffold innovation, poor cross-target generalization capability, and fails to deliver ligand-target interaction details. | [13] |
| ChemMine Tools |  | Fingerprint and embedding/indexing algorithms |  |  |  |
| BRUSELAS |  | 3D shape and pharmacophore searches | Not dependent on specific chemical structures/skeletons, suitable for situations where the target structure is unknown | Ignore chemical characteristics, highly sensitive to conformation | [14] |
| pepMMsMIMIC |  | Multi-conformers 3D-similarity search |  |  | [15] |
| ChemSAR |  | SAR classification models | Quickly construct DL models, de novo generates a chemical library and use those models for VS | Modeling data relies on public databases | [16] |
| DeepScreening |  | Classification and regression models |  |  | [17] |
| OCHEM/MLViS |  | QSAR | Quantitative structure-activity relationship, used when the target structure is unknown, suitable for large-scale screening | Highly dependent on the quality of training data, the contradiction between descriptor selection and model complexity | [18, 19] |
| GeminiMol |  | Capturing the complicated interplay between the molecular structure and the conformational space | Enable training reliable molecular representation models without including experimental molecular properties | Unable to screen compounds with special structures such as covalent compounds and metal ion coordination compounds | [20] |
| ITCM | VS based on multiple omics | Transcriptomics | Multidimensional target identification, enhancing clinical relevance, circumventing structural dependencies, accelerating hit compound discovery | High complexity of data integration, challenges in experimental validation of multi-omics mechanisms, scarcity of omics data for rare diseases, inadequate capture of dynamic biological processes | [21] |
| BATMAN-TCM 2.0 |  | Transcriptomics, genomics, proteomics |  |  | [22] |
| GSFM |  | Transcriptomics, genomics, proteomics |  |  | [23] |
| IGTCM |  | Genomes |  |  | [24] |
| IMP |  | Based on genomes |  |  | [25] |
| HERB |  | Based on transcriptomics |  |  | [26] |
| TCMSP | VS based ADME filtering | MW, HL, OB, DL, BBB pharmacokinetic properties... | Eliminating molecules with poor drug-likeness, significantly reducing costs, computational efficiency, enhanced clinical translation potential | Oversimplification of physiological complexity, highly variable reliability of prediction models, lack of in *vivo* dynamic data, and neglect of transporter interactions | [27] |
| SwissADME |  | Physicochemical properties, pharmacokinetics, drug-likeness and medicinal chemistry friendliness, among which in-house proficient methods |  |  | [28] |
| ADMETlab 3.0 |  | 21 physicochemical properties, 19 medicinal chemistry properties, 34 ADME endpoints, 36 toxicity endpoints, and 8 toxicophore rules |  |  | [29] |
| admetSAR3.0 |  | Over 370 000 high-quality experimental ADMET data for 104 652 unique compounds, 119 endpoints |  |  | [30] |
| OptADMET |  | 41,779 validated transformation rules generated from the analysis of 177,191 reliable experimental datasets; 146,450 rules were generated by analyzing 239,194 molecular data predictions. |  |  | [31] |
| ADMETopt |  | More than 50 000 unique scaffolds, up to 15 ADMET properties |  |  | [32] |
| Interpretable-ADMET |  | 250729 entries associated with 59 kinds of ADMET-associated properties for 80167 chemical compounds. |  |  | [33] |
| BATCHIE | Combination drug screens | Bayesian | Take any Bayesian model and design optimal batches with respect to that model | Limited by the data on which they are trained | [34] |

**Label:** ADMET: Absorption, Distribution, Metabolism, Excretion, Toxicity; MW: Molecular weight; HL:half-life; OB:Oral bioavailability; DL: drug-like; BBB: Blood-brain barrier

**Reference:**

1. Labbé CM, Rey J, Lagorce D, Vavruša M, Becot J, Sperandio O, Villoutreix BO, Tufféry P, Miteva MA: **MTiOpenScreen: a web server for structure-based virtual screening**. *Nucleic Acids Res* 2015, **43**(W1):W448-454.

2. Guedes IA, Pereira da Silva MM, Galheigo M, Krempser E, de Magalhães CS, Correa Barbosa HJ, Dardenne LE: **DockThor-VS: A Free Platform for Receptor-Ligand Virtual Screening**. *J Mol Biol* 2024, **436**(17):168548.

3. Douguet D: **e-LEA3D: a computational-aided drug design web server**. *Nucleic Acids Res* 2010, **38**(Web Server issue):W615-621.

4. Sunseri J, Koes DR: **Pharmit: interactive exploration of chemical space**. *Nucleic Acids Res* 2016, **44**(W1):W442-448.

5. Vangone A, Schaarschmidt J, Koukos P, Geng C, Citro N, Trellet ME, Xue LC, Bonvin A: **Large-scale prediction of binding affinity in protein-small ligand complexes: the PRODIGY-LIG web server**. *Bioinformatics* 2019, **35**(9):1585-1587.

6. London N, Miller RM, Krishnan S, Uchida K, Irwin JJ, Eidam O, Gibold L, Cimermančič P, Bonnet R, Shoichet BK *et al*: **Covalent docking of large libraries for the discovery of chemical probes**. *Nat Chem Biol* 2014, **10**(12):1066-1072.

7. Labbé CM, Pencheva T, Jereva D, Desvillechabrol D, Becot J, Villoutreix BO, Pajeva I, Miteva MA: **AMMOS2: a web server for protein-ligand-water complexes refinement via molecular mechanics**. *Nucleic Acids Res* 2017, **45**(W1):W350-w355.

8. Wang J, Dokholyan NV: **MedusaDock 2.0: Efficient and Accurate Protein-Ligand Docking With Constraints**. *J Chem Inf Model* 2019, **59**(6):2509-2515.

9. Koes DR, Dömling A, Camacho CJ: **AnchorQuery: Rapid online virtual screening for small-molecule protein-protein interaction inhibitors**. *Protein Sci* 2018, **27**(1):229-232.

10. Lee A, Kim D: **CRDS: Consensus Reverse Docking System for target fishing**. *Bioinformatics* 2020, **36**(3):959-960.

11. Zhou G, Rusnac DV, Park H, Canzani D, Nguyen HM, Stewart L, Bush MF, Nguyen PT, Wulff H, Yarov-Yarovoy V *et al*: **An artificial intelligence accelerated virtual screening platform for drug discovery**. *Nat Commun* 2024, **15**(1):7761.

12. Lu JM, Wang HF, Guo QH, Wang JW, Li TT, Chen KX, Zhang MT, Chen JB, Shi QN, Huang Y *et al*: **Roboticized AI-assisted microfluidic photocatalytic synthesis and screening up to 10,000 reactions per day**. *Nat Commun* 2024, **15**(1):8826.

13. Dong J, Cao DS, Miao HY, Liu S, Deng BC, Yun YH, Wang NN, Lu AP, Zeng WB, Chen AF: **ChemDes: an integrated web-based platform for molecular descriptor and fingerprint computation**. *J Cheminform* 2015, **7**:60.

14. Banegas-Luna AJ, Cerón-Carrasco JP, Puertas-Martín S, Pérez-Sánchez H: **BRUSELAS: HPC Generic and Customizable Software Architecture for 3D Ligand-Based Virtual Screening of Large Molecular Databases**. *J Chem Inf Model* 2019, **59**(6):2805-2817.

15. Floris M, Masciocchi J, Fanton M, Moro S: **Swimming into peptidomimetic chemical space using pepMMsMIMIC**. *Nucleic Acids Res* 2011, **39**(Web Server issue):W261-269.

16. Dong J, Yao ZJ, Zhu MF, Wang NN, Lu B, Chen AF, Lu AP, Miao H, Zeng WB, Cao DS: **ChemSAR: an online pipelining platform for molecular SAR modeling**. *J Cheminform* 2017, **9**(1):27.

17. Liu Z, Du J, Fang J, Yin Y, Xu G, Xie L: **DeepScreening: a deep learning-based screening web server for accelerating drug discovery**. *Database (Oxford)* 2019, **2019**.

18. Sushko I, Novotarskyi S, Körner R, Pandey AK, Rupp M, Teetz W, Brandmaier S, Abdelaziz A, Prokopenko VV, Tanchuk VY *et al*: **Online chemical modeling environment (OCHEM): web platform for data storage, model development and publishing of chemical information**. *J Comput Aided Mol Des* 2011, **25**(6):533-554.

19. Korkmaz S, Zararsiz G, Goksuluk D: **MLViS: A Web Tool for Machine Learning-Based Virtual Screening in Early-Phase of Drug Discovery and Development**. *PLoS One* 2015, **10**(4):e0124600.

20. Wang L, Wang S, Yang H, Li S, Wang X, Zhou Y, Tian S, Liu L, Bai F: **Conformational Space Profiling Enhances Generic Molecular Representation for AI-Powered Ligand-Based Drug Discovery**. *Adv Sci (Weinh)* 2024, **11**(40):e2403998.

21. Tian S, Zhang J, Yuan S, Wang Q, Lv C, Wang J, Fang J, Fu L, Yang J, Zu X *et al*: **Exploring pharmacological active ingredients of traditional Chinese medicine by pharmacotranscriptomic map in ITCM**. *Brief Bioinform* 2023, **24**(2).

22. Kong X, Liu C, Zhang Z, Cheng M, Mei Z, Li X, Liu P, Diao L, Ma Y, Jiang P *et al*: **BATMAN-TCM 2.0: an enhanced integrative database for known and predicted interactions between traditional Chinese medicine ingredients and target proteins**. *Nucleic Acids Res* 2024, **52**(D1):D1110-d1120.

23. Tian S, Liao X, Cao W, Wu X, Chen Z, Lu J, Wang Q, Zhang J, Chen L, Zhang W: **GSFM: A genome-scale functional module transformation to represent drug efficacy for in silico drug discovery**. *Acta Pharmaceutica Sinica B* 2024.

24. Ye YN, Liang DF, Yi JH, Jin S, Zeng Z: **IGTCM: An integrative genome database of traditional Chinese medicine plants**. *Plant Genome* 2023, **16**(2):e20317.

25. Chen T, Yang M, Cui G, Tang J, Shen Y, Liu J, Yuan Y, Guo J, Huang L: **IMP: bridging the gap for medicinal plant genomics**. *Nucleic Acids Res* 2024, **52**(D1):D1347-d1354.

26. Fang S, Dong L, Liu L, Guo J, Zhao L, Zhang J, Bu D, Liu X, Huo P, Cao W *et al*: **HERB: a high-throughput experiment- and reference-guided database of traditional Chinese medicine**. *Nucleic Acids Res* 2021, **49**(D1):D1197-d1206.

27. Ru J, Li P, Wang J, Zhou W, Li B, Huang C, Li P, Guo Z, Tao W, Yang Y *et al*: **TCMSP: a database of systems pharmacology for drug discovery from herbal medicines**. *J Cheminform* 2014, **6**:13.

28. Daina A, Michielin O, Zoete V: **SwissADME: a free web tool to evaluate pharmacokinetics, drug-likeness and medicinal chemistry friendliness of small molecules**. *Sci Rep* 2017, **7**:42717.

29. Fu L, Shi S, Yi J, Wang N, He Y, Wu Z, Peng J, Deng Y, Wang W, Wu C *et al*: **ADMETlab 3.0: an updated comprehensive online ADMET prediction platform enhanced with broader coverage, improved performance, API functionality and decision support**. *Nucleic Acids Res* 2024, **52**(W1):W422-w431.

30. Gu Y, Yu Z, Wang Y, Chen L, Lou C, Yang C, Li W, Liu G, Tang Y: **admetSAR3.0: a comprehensive platform for exploration, prediction and optimization of chemical ADMET properties**. *Nucleic Acids Res* 2024, **52**(W1):W432-w438.

31. Yi J, Shi S, Fu L, Yang Z, Nie P, Lu A, Wu C, Deng Y, Hsieh C, Zeng X *et al*: **OptADMET: a web-based tool for substructure modifications to improve ADMET properties of lead compounds**. *Nat Protoc* 2024, **19**(4):1105-1121.

32. Yang H, Sun L, Wang Z, Li W, Liu G, Tang Y: **ADMETopt: A Web Server for ADMET Optimization in Drug Design via Scaffold Hopping**. *J Chem Inf Model* 2018, **58**(10):2051-2056.

33. Wei Y, Li S, Li Z, Wan Z, Lin J: **Interpretable-ADMET: a web service for ADMET prediction and optimization based on deep neural representation**. *Bioinformatics* 2022, **38**(10):2863-2871.

34. Tosh C, Tec M, White JB, Quinn JF, Ibanez Sanchez G, Calder P, Kung AL, Dela Cruz FS, Tansey W: **A Bayesian active learning platform for scalable combination drug screens**. *Nat Commun* 2025, **16**(1):156.
