# Supplementary material for: Advancing the modernization of traditional Chinese medicine through artificial intelligence and multimodal data integration
Source: Chin Med. 2026 Jan 26;21:54. doi: 10.1186/s13020-025-01194-y (PMC12833950; doi:10.1186/s13020-025-01194-y)
Supplement: Supplementary file 4 — Supplementary Material 4 [file 13020_2025_1194_MOESM4_ESM.docx]

**Table 9.** Introduction of existing LLMs for TCM

| **TCM large language models** | **Full name** | **Foundation model** | **Instruction data** | **Training paradigm** | **Model evaluation** | **Scenarios** | **Ref.** |
| --- | --- | --- | --- | --- | --- | --- | --- |
| TCMChat | LLMs | Baichuan2-7B-Chat | 4 national standards, 7 medical textbooks, 18 medical cases | PT, SFT | Accuracy, BLEU, Meteor, ROUGE-1, ROUGE-2, ROUGE-L, BertScore | TCM knowledgebase, choice question, reading comprehension, entity extraction, medical case diagnosis, and herb or formula recommendation | [1] |
| Lingdan | Lingdan-TCPM-Chat and Lingdan-PR | Baichuan2-13B-Base | TCM ancient books, textbooks, and clinical data | PT, SFT | F1-score | Q&A on TCM Clinical Knowledge and Recommendations for Herbal Prescriptions | [2] |
| MedChatZH | Large Language Model Meta AI | Baichuan-7B | 1000 TCM books | PT, FT | BLEU, GLEU, ROUGE-1, ROUGE-2,  and ROUGE-L | Q&A and dialogue systems | [3] |
| OpenTCM |  | GraphRAG-empowered | 68 gynecological books | PT, FT | Precision, Recall, F1-Score, Accuracy | TCM information search and intelligent Q&A | [4] |
| CPMI-ChatGLM |  | ChatGLM-6B | 3906 annotated Chinese patent drug records | FT | BLEU, ROUGE, and BART Score | TCM recommendations and medication suggestions | [5] |
| ACUBERT |  | BERT model | 54593 different entities from 82 acupuncture medical books | SVM, RF | Precision, recall, F1 scores | Meridian entity recognition and classification | [6] |
| JingFang |  | Qwen2.5-7B-Instruct and the Roberta model | Over 63,000 real diagnostic data entries to select over 43,000 high-quality data entries | FT | Weighted precision, weighted recall, weighted F1 | Expert-level medical diagnosis and syndrome differentiation-based treatment | https://arxiv.org/abs/2502.04345 |
| ZhongJing TCM model |  | LLaMA | Proprietary medical dataset | PT, SFT, RLHF |  | TCM Q&A |  |
| Huang-Di model warehouse |  | Ziya-LLaMA-13B-V1 | TCM ancient Books, TCM websites | PT, FT, DPO |  | Q&A on TCM ancient books knowledge | https://github.com/Zlasejd/HuangDI |
| ShenNong-TCM-LLM | ShenNong-TCM-LLM | LlaMA | TCM classics from ancient to modern Times | LoRA FT (rank=16) |  | Inheritance of TCM | https://github.com/michael-wzhu/ShenNong-TCM-LLM |

**Label:** DPO: Direct preference optimization; LLMs: Ground-breaking large language models; RLHF: Reinforcement learning from human feedback; PT: pre-trained; FT: fine-tuned; SFT: Supervised Fine-Tuning; RLHF: Reinforcement learning from human feedback; Q&A: Question-Answering; BLEU: Bilingual evaluation understudy; Meteor: Metric for evaluation of translation with explicit ordering; ROUGE-1/2/L: Recall-oriented understudy for gisting evaluation; BertScore: BERT-based evaluation score; SVM: support vector machine; RF: random forest

**Reference:**

1. Dai Y, Shao X, Zhang J, Chen Y, Chen Q, Liao J, Chi F, Zhang J, Fan X: **TCMChat: A generative large language model for traditional Chinese medicine**. *Pharmacol Res* 2024, **210**:107530.

2. Hua R, Dong X, Wei Y, Shu Z, Yang P, Hu Y, Zhou S, Sun H, Yan K, Yan X *et al*: **Lingdan: enhancing encoding of traditional Chinese medicine knowledge for clinical reasoning tasks with large language models**. *J Am Med Inform Assoc* 2024, **31**(9):2019-2029.

3. Tan Y, Zhang Z, Li M, Pan F, Duan H, Huang Z, Deng H, Yu Z, Yang C, Shen G *et al*: **MedChatZH: A tuning LLM for traditional Chinese medicine consultations**. *Comput Biol Med* 2024, **172**:108290.

4. He J, Guo Y, Lam LK, Leung W, He L, Jiang Y, Chiu Wang C, Xing G, Chen H: **OpenTCM: A GraphRAG-Empowered LLM-based System for Traditional Chinese Medicine Knowledge Retrieval and Diagnosis**. In*.*; 2025: arXiv:2504.20118.

5. Liu C, Sun K, Zhou Q, Duan Y, Shu J, Kan H, Gu Z, Hu J: **CPMI-ChatGLM: parameter-efficient fine-tuning ChatGLM with Chinese patent medicine instructions**. *Sci Rep* 2024, **14**(1):6403.

6. Xu T, Wen J, Wang L, Huang Y, Zhu Z, Zhu Q, Fang Y, Yang C, Xia Y: **Acupuncture indication knowledge bases: meridian entity recognition and classification based on ACUBERT**. *Database (Oxford)* 2024, **2024**.
