# Supplementary material for: Advancing the modernization of traditional Chinese medicine through artificial intelligence and multimodal data integration
Source: Chin Med. 2026 Jan 26;21:54. doi: 10.1186/s13020-025-01194-y (PMC12833950; doi:10.1186/s13020-025-01194-y)
Supplement: Supplementary file 5 — Supplementary Material 5 [file 13020_2025_1194_MOESM5_ESM.docx]

**Supplementary Material to Reviewers**

We are truly grateful to the reviewers for their careful analysis of our manuscript entitled “**Advancing the modernization of traditional Chinese medicine through artificial intelligence and multimodal data integration**”. We would like to express our appreciation for their insight, pertinent remarks and thoughtful suggestions on our manuscript. We have carefully studied the **3^st^ Reviewer** comments, and the manuscript has been revised accordingly. We hope our revision can help editors and reviewers endorse the quality of this research.

**Responses to 3^st^ Reviewer comments**

Reviewer: The current Fig.2 should be optimized to enhance the clarity of the screening process. The logical progression of screening should be more clearly illustrated to help readers better understand each step. Consider providing a more accurate breakdown of the numbers in each stage, ensuring consistency in the figures presented (e.g., the discrepancy between '3907 records screened' and '245 records remaining'). Additionally, labeling or briefly explaining key decisions or criteria at each stage would help readers follow the screening rationale more easily. This way, the overall flow and reasoning behind the screening will be clearer and more accessible to the reader.

**Response**: Thank you for your professional editorial feedback. We have provided a detailed explanation of the screening process for each stage as requested, and the revised sections can be found on **page 7** of the manuscript.
